# Targeting macrophage migration inhibitory factor as a potential therapeutic strategy in colorectal cancer

**DOI:** 10.1038/s41389-025-00572-3

**Published:** 2025-08-20

**Authors:** Kim Lucia Schneider, Luisa Claus, Richard Bucala, Ramona Schulz-Heddergott

**Affiliations:** 1 Department of Molecular Oncology, University Medical Center Göttingen, Göttingen, Germany; 2 Departments of Medicine, Pathology, and Epidemiology & Public Health, Yale School of Medicine and Yale Cancer Center, New Haven, CT USA

**Keywords:** Colorectal cancer, Prognostic markers

## Abstract

Survival rates for patients with late-stage colorectal cancer (CRC) remain low due to limited efficacy of current therapeutic regimens. To overcome these challenges, novel drug targets are urgently needed. Macrophage migration inhibitory factor (MIF), an upstream immunoregulatory cytokine, has emerged as a potential target due to its multifaceted role in cancer pathogenesis. During tumorigenesis, MIF protein levels are often elevated in tumor cells through chaperone-mediated stabilization. Although several in vivo studies have implicated MIF in tumor initiation and progression, its role in sustaining established tumors, particularly when derived from epithelial tumor cells, remained unclear. Using a constitutive *Mif* knockout mouse model, we previously demonstrated that MIF is required for CRC development. Now, we expanded our experimental CRC model towards a more therapeutic rationale. We hypothesized that epithelial-derived MIF is essential for tumor maintenance and might serve as a possible cancer drug target. Therefore, we depleted epithelial MIF during late-stage CRC tumorigenesis in two genetically-engineered and chemically-induced murine CRC models. Our proof-of-principle study reveals that *Mif* depletion in epithelial tumor cells attenuates cancer maintenance in both CRC models, coinciding with reduced macrophage recruitment and angiogenesis. Our data highlight the potential utility of targeting MIF in CRC patients for therapeutic benefit.

## Introduction

Colorectal cancer (CRC) is the third most common cancer worldwide (https://seer.cancer.gov/statfacts/html/colorect.html) [1]. The majority of CRC patients (75%) harbor non-hereditary, sporadic CRC. Patient survival strongly depends on the early detection of low-stage CRC which is improved by screening and surgery options [2]. However, late-stage CRC patient survival is still low. The relative 5-year survival rate with stage 3 CRC is ~65% and with stage 4 CRC ~10% [3]. To address therapeutic challenges in late-stage CRC, new approaches are needed and involve the targeting of tumor cell specific drivers. Macrophage migration inhibitory factor (MIF) might serve as specific drug target.

MIF, an immunoregulatory cytokine, plays multiple roles in the pathogenesis of cancer and inflammation [4–15]. In CRC, MIF is aberrantly elevated in tumor cells and is associated with poorer survival outcomes and reduced responses to therapy [16–18]. Clinical studies have shown that CRC patients with high MIF expression, particularly in combination with its receptor CD74, exhibit the shortest overall survival [18, 19]. The tumor specificity of MIF mainly arises from its overexpression and/or chaperone-mediated stabilization, particularly in tumor epithelial cells [18, 20, 21], making it to an attractive therapeutic target. In brief, we previously identified that MIF binds to the molecular chaperone heat shock protein 90 (HSP90), resulting in hyperstabilization of MIF in epithelial cells, including colon cancer cells [18, 20, 21]. This stabilization is predominantly tumor-specific, as it occurs in epithelial tumor cells but not in stromal or inflammatory cells. The stabilization of tumor-promoting proteins by the HSP90 chaperone system is a hallmark of tumorigenesis, particularly in malignant tumors of epithelial origin [22–26]. Moreover, HSP90-stabilized proteins, such as MIF, are secreted into the tumor microenvironment, where they further support tumor progression [18, 27, 28]. Under normal physiological conditions, heat shock proteins (HSPs) assist in the proper folding of proteins, promote optimal activation of e.g. transcription factors, and maintenance of proteasome function. Misfolded or irreversibly damaged proteins are typically targeted for degradation by chaperones. Importantly, during oncogenesis, this regulatory system is hijacked. Cancer-associated stresses - such as proteotoxic stress, hypoxia, and nutrient deprivation - lead to the hyperactivation of heat shock factor 1 (HSF1), the master transcription factor of HSPs [22, 29–34]. This results in overexpression of HSPs, which support the malignant phenotype by stabilizing oncogenic and tumor-promoting proteins, including kinases, transcription factors, and cytokines like MIF [21, 22, 33, 35]. Consequently, cancer cells become dependent on this chaperone network, creating a cancer-specific vulnerability and presenting a promising therapeutic opportunity.

Elevated MIF levels have been shown in various human cancer entities such as breast, ovary, colon, prostate, liver, lung, pituitary and brain [14]. In vivo studies using constitutive mouse models further support MIF’s role in cancer progression, with evidence from breast, skin, pancreas, intestine, and bladder cancers [17, 18, 20, 36–40]. Thereby, MIF exerts its effects not only within epithelial tumor cells but also in stromal fibroblasts and immune cells of the tumor microenvironment [41–44]. However, aberrant MIF expression within tumor cells has emerged as a key driver of tumorigenesis, contributing to multiple oncogenic pathways [12, 14, 15, 45, 46]. In CRC, there is strong evidence for a positive correlation between MIF levels and disease progression. Elevated MIF in serum and tumor tissue correlates with tumor differentiation, lymph node involvement, and liver metastasis in CRC patients [16, 47]. MIF is consistently overexpressed in both tumor tissues and sera of patients with primary sporadic CRC [17, 48]. Furthermore, MIF’s classical receptor, CD74, which promotes cancer cell proliferation, is increasingly expressed during the transition from colitis to adenoma and carcinoma in human tissues [49, 50]. Functionally, MIF has been shown to promote tumor growth in preclinical models. In an intestinal tumor model, constitutive *Mif* deletion in *Apc*^MIN/+^ mice reduced adenoma number and size [17]. We further demonstrated in a CRC mouse model with constitutive *Mif* deletion that MIF is specifically stabilized in tumors and promotes colorectal tumor growth [18]. Additionally, the genetic MIF-173G/C polymorphisms of MIF is associated with CRC stage progression [51, 52], and has been linked to susceptibility and phenotype of inflammatory bowel diseases such as Crohn’s disease [53, 54]. Despite numerous correlations between aberrant MIF and CRC progression, its causative role in established tumors remains untested. Regarding anti-cancer therapies, it is still unclear whether fully developed tumors are dependent on MIF for maintenance. Although some MIF-targeting therapies have been evaluated in CRC models with established tumors and have shown mild to moderate efficacy [16, 55], most in vivo studies using genetically modified mice to date have been conducted in a preventative setting with constitutive *Mif* knockout models [17, 18, 39, 56], or in a hepatocyte-specific *Mif* deletion initiated before tumor formation [57]. To rigorously assess MIF’s role in tumor maintenance, inducible and tissue-specific genetic models are needed. Such models would enable the temporal inactivation of MIF after tumor initiation, providing critical proof-of-principle for its functional relevance in sustaining tumor growth. Additionally, it remains to be clarified which cellular sources of MIF - tumor epithelial cells, stromal cells, immune cells, or a combination - are critical for CRC maintenance.

To advance our experimental CRC model towards a more therapeutically relevant setting, we investigated inducible, tissue-specific *Mif* ablation in two genetically engineered and chemically induced CRC mouse models. To mimic distinct stages of CRC progression, we employed two *TP53*-based mouse models, each carrying the humanized missense mutation *TP53*^R248Q^, which is known to enhance tumor aggressiveness. The first model is heterozygous for the *TP53*^R248Q^ allele (*TP53*^Q/+^) predominantly developing adenomas, while the second model harbors homozygous *TP53*^R248Q^ alleles (*TP53*^Q/Q^) exhibiting gain-of-function phenotypes that promote increased tumor proliferation and invasiveness [58–61]. Both models additionally harbor floxed *Mif* (*Mif*^fl/fl^) alleles and express a tamoxifen-inducible *cre* recombinase under the control of the villin promoter (*villinCreER*^T2^), enabling time- and tissue-specific *Mif* deletion in intestinal epithelial cells (*Mif*^∆IEC^). Colorectal tumors were induced using the chemical carcinogenesis protocol with azoxymethane (AOM) and dextran sodium sulfate (DSS). Following the development of established tumors, tamoxifen (TAM) administration activated Cre recombinase, leading to recombination and deletion of the *Mif* allele specifically in tumor epithelial cells. Strikingly, the inducible depletion of MIF significantly reduced tumor growth. This was accompanied by decreased tumor cell proliferation, reduced macrophage infiltration into the tumor bulk, and impaired tumor-associated angiogenesis. These proof-of-principle experiments demonstrate that established colorectal tumors are critically addicted on stabilized, tumor cell-intrinsic MIF - even in aggressive, malignant stages. Consequently, MIF represents a selective and actionable therapeutic target, highlighting its potential as a promising opportunity for anti-cancer therapies.

## Results

### Epithelial tumor-derived MIF is essential for maintenance of colorectal adenomas

Previously, we have shown a tumor-specific MIF stabilization in the murine AOM/DSS colorectal tumor model. Furthermore, a classical, constitutive *Mif* knock-out led to a reduced tumor growth [18]. Nevertheless, using such constitutive *Mif* knock-out mouse, tumors including stromal cells nevertheless grew without MIF from the state of initiation. That led us to the more therapeutic question of whether acute ablation of epithelial MIF in established tumors might also reduce tumor growth and consequently, whether established tumors require epithelial MIF.

**Fig. 1:**
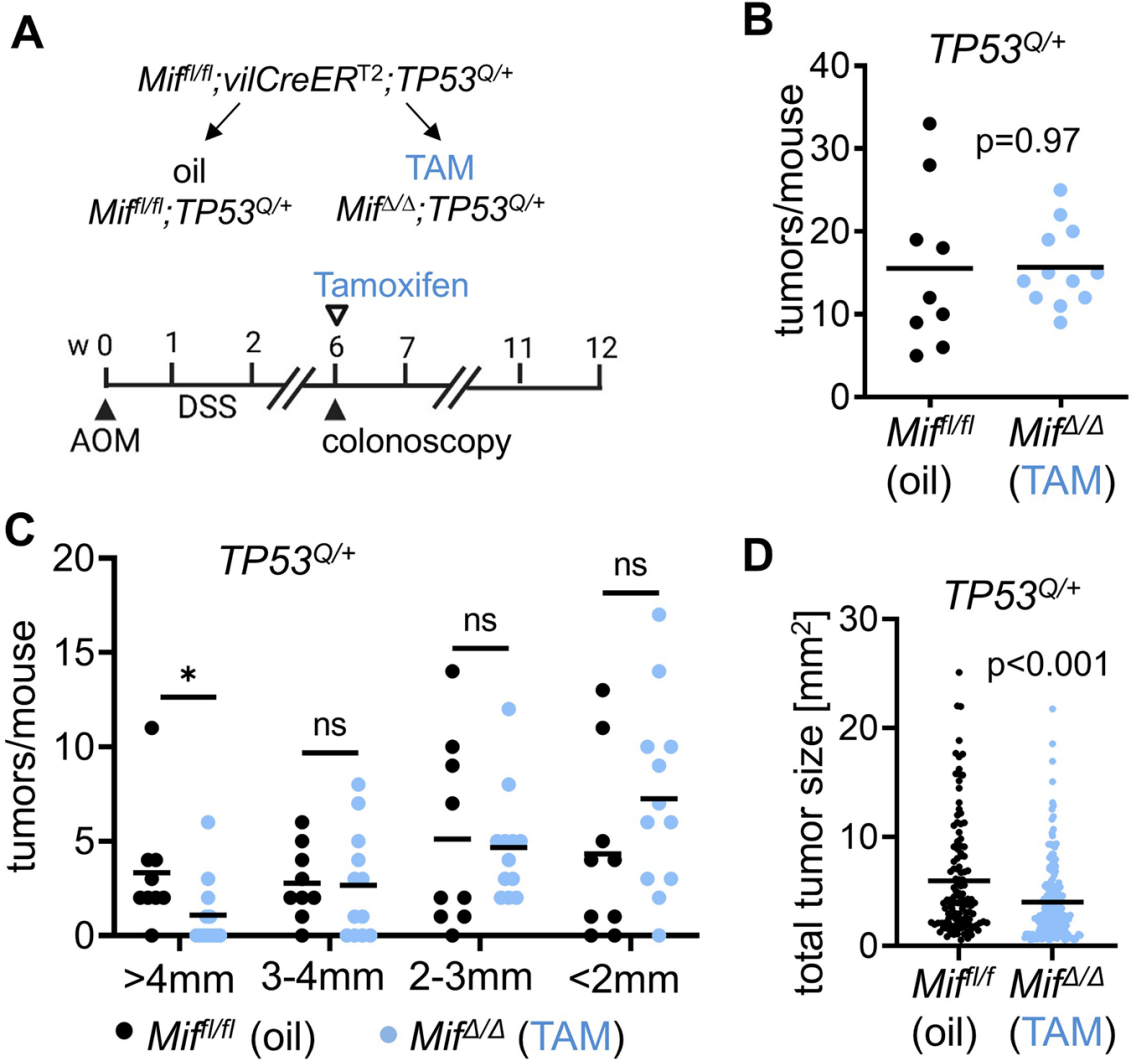
Epithelial tumor-derived MIF is essential for maintenance of murine colorectal adenomas. **A** Scheme of colorectal tumor induction via AOM and DSS, and induction of time-dependent *Mif* recombination in epithelial tumor cells via Tamoxifen. The floxed *Mif* alleles (*Mif*^*fl/fl*^) were paired with inducible *CreER*^T2^ alleles under control of the villin promotor (*vilCreER*^T2^) in a heterozygous, humanized *TP53*^R248Q^ (*TP53*^*Q/+*^) background. After tumor initiation, tumor development was visualized by colonoscopy once per week, starting at week 6. Once tumors were established, mice were treated with Tamoxifen (TAM) or vehicle (oil). Tumors were analyzed at 12 weeks post-AOM. Oil-treated *Mif*^*fl/fl*^;*vilCreER*^*T*2^;*TP53*^*Q/+*^ mice are abbreviated as *Mif*^*fl/fl*^;*TP53*^*Q/+*^ and if TAM-treated as *Mif*^*∆/∆*^;*TP53*^*Q/+*^. **B** Analysis of total tumor number per mouse of oil-treated *Mif*^*fl/fl*^;*TP53*^*Q/+*^ (oil) and TAM-treated *Mif*^*∆/∆*^;*TP53*^*Q/+*^ (TAM) mice at 12 weeks post-AOM injection. *Mif*^*fl/fl*^;*TP53*^*Q/+*^ mice: *n* = 9; mean = 15.6. *Mif*^*∆/∆*^;*TP53*^*Q/+*^ mice: *n* = 12; mean = 15.7. **C** Macroscopic analysis of separated tumor sizes per mouse of *Mif*^*fl/fl*^;*TP53*^*Q/+*^ (oil) and *Mif*^*∆/∆*^;*TP53*^*Q/+*^ (TAM) mice at 12 weeks post-AOM injection. *Mif*^*fl/fl*^;*TP53*^*Q/+*^ mice: *n* = 9; mean = 3.3 (4 mm), 2.8 (3–4 mm), 5.1 (2–3 mm), 4.3 (2 mm). *Mif*^*∆/∆*^;*TP53*^*Q/+*^ mice: *n* = 12; mean = 1.1 (4 mm), 2.7 (3–4 mm), 4.7 (2–3 mm), 7.3 (2 mm). ns not significant; *p ≤ 0.05. **D** Total tumor sizes of macroscopically analyzed oil-treated *Mif*^*fl/fl*^;*TP53*^*Q/+*^ and TAM-treated *Mif*^*∆/∆*^;*TP53*^*Q/+*^ mice at 12 weeks post-AOM injection. *Mif*^*fl/fl*^;*TP53*^*Q/+*^ group: *n* = 122 tumors out of 9 mice; mean = 6. *Mif*^*∆/∆*^;*TP53*^*Q/+*^ group: *n* = 188 tumors out of 12 mice; mean = 4. **B**–**D** Student’s *t* test, two-sided. Black lines, mean. **A**–**C** Student’s *t* test, two-sided. Black lines, mean.

Therefore, we sought to mimic the clinical situation and depleted MIF during tumorigenesis. We crossed mice with floxed *Mif* alleles (*Mif*^fl/fl^) to *villinCreER*^T2^ mice (*vilCreER*^*T2*^) to generate Tamoxifen (TAM) – inducible MIF deletions (*Mif*^∆/∆^) restricted to intestinal epithelial cells (Fig. 1A). *Mif*^*fl/fl*^*;vilCreER*^*T2*^ mice were further combined with one humanized constitutive *TP53*^R248Q^ gain-of-function (GOF) allele (*TP53*^*Q/+*^). In principle, complete inactivation of the tumor suppressor *TP53* (p53) by mutations and p53 loss-of-heterozygosity is a hallmark event during tumorigenesis and affects tumor biology by increasing cell proliferation, invasion and therapy resistances [62–65]. In contrast, heterozygous *TP53* hotspot mutation can partially accelerate tumorigenesis and serves for less aggressive tumor models with moderate proliferation and rare pre-invasive tumors [59, 60, 66]. In the AOM/DSS mouse model, heterozygous *TP53*^*Q/+*^ tumors are non-invasive with residual transcriptional p53 activity [58, 59], and thus, served as model for benign polypoid, low-stage tumors. *Mif*^*fl/fl*^;*vilCreER*^*T*2^;*TP53*^*Q/+*^ mice were subjected to AOM/DSS and tumor growth was visualized via colonoscopy (Fig. 1A). At a defined tumor burden, mainly at week 6 after AOM induction, genetic ablation of epithelial MIF was induced by TAM (abbreviated by *Mif*^*Δ/Δ*^*;TP53*^*Q/+*^*)*. Controls were oil-treated *Mif*^*fl/fl*^;*vilCreER*^*T*2^;*TP53*^*Q/+*^ mice, abbreviated by *Mif*^*fl/fl*^*;TP53*^*Q/+*^ from here on (Fig. 1A).

As expected, the total tumor numbers per mouse had not changed 6 weeks after TAM-induced MIF ablation, because MIF was depleted in established tumors (Fig. 1B). Consequently, total tumors were further grouped in different sizes (Fig. 1C). Interestingly, at 6 weeks post TAM, larger tumors were significantly reduced after MIF ablation, compared to the oil-treated *Mif*^*fl/fl*^*;TP53*^*Q/+*^ group (Fig. 1C). In line, calculation of the total tumor area confirmed that a MIF depletion reduced the growth of established tumors (Fig. 1D). With additional control groups, we analyzed whether Tamoxifen itself or its induced Cre activation might have unspecific effects in our model system. Therefore, unfloxed *Mif* alleles (*Mif*^*+l+*^) were combined with *vilCreER*^*T*2^ and *TP53*^*Q/*+^ alleles (Supplementary Fig. S1). Resulting *Mif*^*+/+*^;*vilCreER*^*T*2^;*TP53*^*Q/+*^ mice were treated with AOM/DSS and oil or TAM as *Mif*^*fl/fl*^*;TP53*^*Q/+*^ mouse groups (Fig. 1A). Neither total tumors per mouse, sizes of tumors nor tumor areas were changed upon TAM treatment compared to oil treatment which excluded nonspecific TAM effects on tumor growth (Supplementary Fig. S1A–C).

**Fig. 2:**
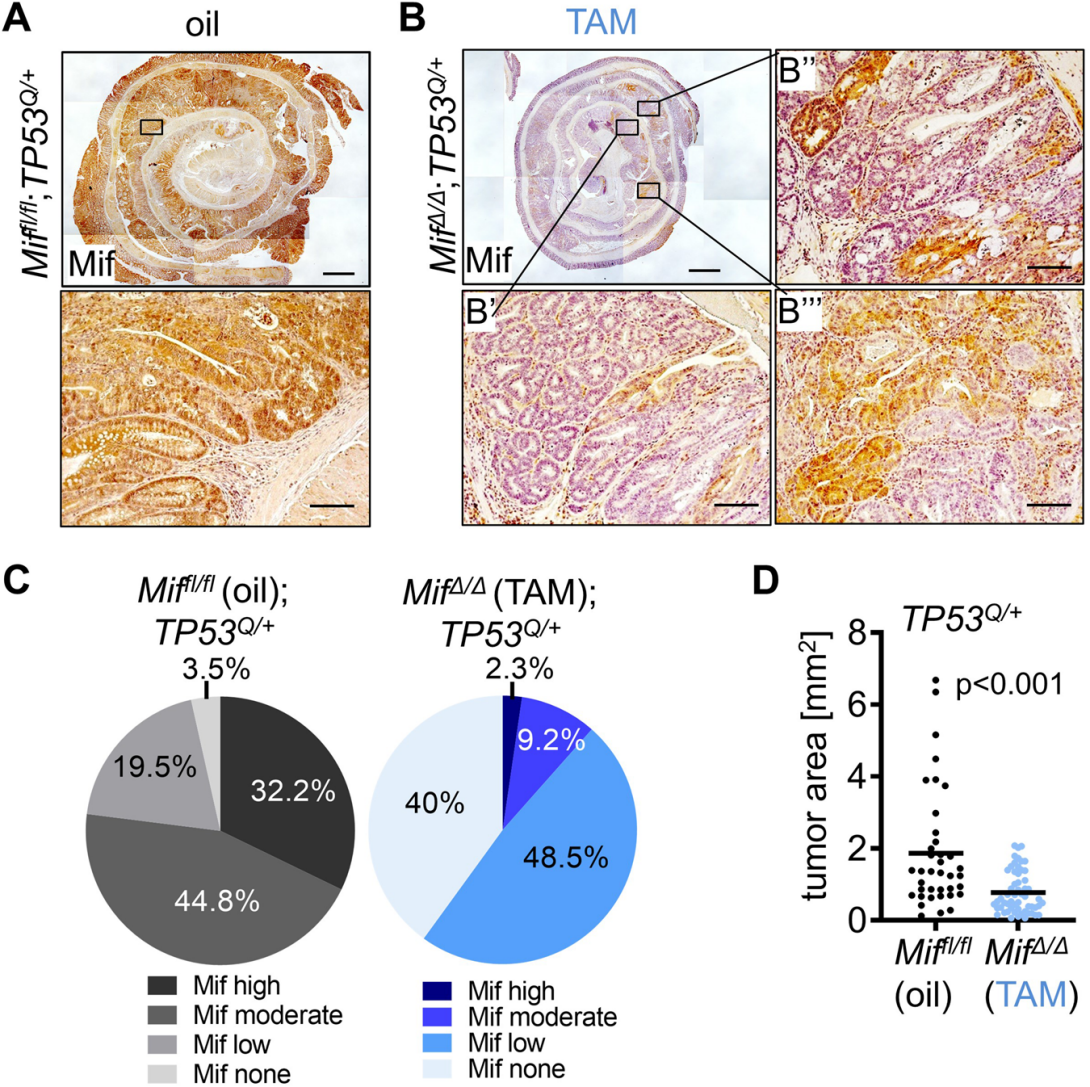
Sufficient *Mif* recombination and Mif protein depletion reduces tumor growth in colorectal adenomas. **A** Representative immunohistological Mif staining in a colonic swiss role (top) and in a higher magnification of a colonic tumor (bottom) from oil-treated *Mif*^*fl/fl*^;*TP53*^*Q/+*^ mice at endpoint 12 weeks post-AOM. Top, rectangle represents the area of the higher magnification (bottom). Scale bar (top), 1000 µm. Scale bar (bottom), 100 µm. **B**–**B”’** Representative immunhistological Mif staining in colonic tissue from TAM-treated *Mif*^*∆/∆*^;*TP53*^*Q/+*^ mice at endpoint. **B** Overview of a colonic swiss roll. Rectangles represent areas of higher magnifications (**B’**–**B”’**). Scale bar, 1000 µm. **B’** No Mif staining in epithelial cells represents efficient recombination. **B”** Low epithelial Mif staining represents acceptable recombination. **B”’** Moderate Mif staining represents inefficient recombination. **B’**–**B”’** Scale bars, 100 µm. **C** Quantification of immunhistological Mif staining of all oil-treated *Mif*^*fl/fl*^;*TP53*^*Q/+*^ (**A**) and all TAM-treated *Mif*^*∆/∆*^;*TP53*^*Q/+*^ (**B**) swiss roles at endpoint. High and moderate Mif staining represents inefficient recombination. No and low Mif staining represents efficient recombination. *Mif*^*fl/fl*^;*TP53*^*Q/+*^ (oil) group: *n* = 87 tumors out of 9 mice. *Mif*^*∆/∆*^ (TAM) *n* = 130 tumors out of 12 mice. **D** Tumor areas of randomly selected *Mif*^*fl/fl*^;*TP53*^*Q/+*^ (oil) tumors and *Mif*^*∆/∆*^;*TP53*^*Q/+*^ (TAM) tumors graded with efficient *Mif* recombination from (C). *Mif*^*fl/fl*^;*TP53*^*Q/+*^ (oil) mice: *n* = 38 tumors out of 7 mice; mean = 1.9. *Mif*^*∆/∆*^;*TP53*^*Q/+*^ (TAM) *n* = 57 tumors out of 8 mice; mean = 0.8. Student’s *t* test, two-sided. Black lines, mean.

Another important point in genetically inducible recombination systems is that Cre recombinase-mediated deletions of alleles are never 100% efficient which might diminish effects of macroscopic analyses (Fig. 1). If MIF is partially retained in tumors, it might affect surrounding tumor cells including the tumor microenvironment through paracrine actions. To analyze the efficiency of *Mif* recombination, we immunostained oil-treated *Mif*^*fl/fl*^*;TP53*^*Q/+*^ and TAM-treated *Mif*^*Δ/Δ*^*;TP53*^*Q/+*^ groups for MIF level at 12 weeks endpoint. As expected, oil-treated *Mif*^*fl/fl*^*;TP53*^*Q/+*^ tumors exhibited a strong and equal MIF protein stabilization in the epithelial tumor part (Fig. 2A). In contrast, TAM-treated *Mif*^*Δ/Δ*^*;TP53*^*Q/+*^ tumors showed a partial and irregular lack of MIF (Fig. 2B). In consequence, we graded the TAM-induced group for MIF level to exclude tumors with insufficient *Mif* recombination (Fig. 2B) in Mif none (Fig. 2B’), Mif low (Fig. 2B”), Mif moderate (Fig. 2B”’) and high Mif level. In 88.5% of TAM-treated tumors, MIF ablation was graded as complete (Mif none or low) and such tumors were used for further microscopic analysis (Fig. 2C). TAM-treated tumors with moderate to high Mif level (11.5%) were excluded. In contrast, in 77% of oil-treated tumors Mif level were graded as high or moderate (Fig. 2C). Indeed, tumor areas of TAM-treated tumors with a sufficient MIF ablation showed a strong reduction compared to oil-treated controls (Fig. 2D). Please note that analysis of sufficient MIF ablations exposed stronger effects with a 57.9% reduction on tumor growth (Fig. 2D) than TAM-treated tumors in the macroscopic analysis with a reduction of 33.3% (Fig. 1D) compared to each oil groups. Again, to address TAM-induced unspecific effects, unfloxed *Mif*^*+l+*^*;TP53*^*Q/+*^ tumors, oil- or TAM-treated, were also immunohistological analyzed for MIF level (Supplementary Fig. S2A). As expected, both groups showed a strong epithelial MIF staining in the majority of tumors. After grading Mif level in TAM-treated *Mif*^*+l+*^*;TP53*^*Q/+*^ tumors, MIF retained with moderate to high level in 89.1% of samples (Supplementary Fig. S2B) which is comparable to oil-treated groups. Furthermore, TAM treatment in *Mif*^*+l+*^*;TP53*^*Q/+*^ tumors failed to reduce tumor areas compared to oil treatment (Supplementary Fig. S2C).

In sum, this proof-of-principle experiment showed that epithelial, stabilized MIF is essential for tumor maintenance. Thus, MIF can serve as a clinically relevant drug target in low-stage colorectal adenomas.

### A genetic MIF ablation reduces established tumor growth in an aggressive colorectal cancer mouse model

To create malignant tumors and to mimic different stages of CRC, we used a second mouse model harboring homozygous *TP53*^R248Q^ GOF alleles (*TP53*^Q/Q^) which triggers an invasive and higher proliferative tumor stage [58, 60]. In detail, we and others discovered that homozygous or hemizygous mutated *TP53* GOF alleles increases tumor growth and invasiveness in different mouse models [66–69]. Mechanistically, in CRC, p53^R248Q^ mutants (mutp53^R248Q^) constitutively hyperactivates STAT3 signaling by forming a physical complex [58]. Genetic ablation of mutp53^R248Q^ strongly reduced tumor growth and invasion in established murine CRC tumors. Importantly, the loss of the wildtype *TP53* allele after the *TP53*^R248Q^ mutagenesis of the first allele (p53 loss-of-heterozygosity = *TP53*^Q*/−*^) or homozygous *TP53*^*Q/Q*^ is a prerequisite for mutp53^R248Q^ gain-of-functions to drive tumor aggressiveness in CRC [59]. Thus, we recapitulated the adenoma–carcinoma pathway to progress aggressive colorectal tumors by engineering homozygous *TP53*^*Q/Q*^ mouse cohorts [62].

**Fig. 3:**
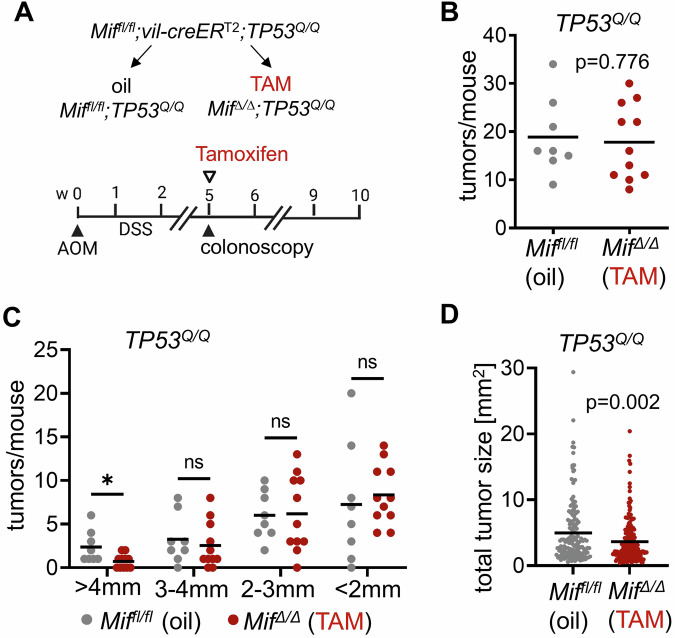
Malignant colorectal tumors are addicted to stabilized, tumor cell-specific MIF. **A** Schematic overview of colorectal tumor initiation in mutant p53 gain-of-function *Mif*^*fl/fl*^;*vilCreER*^*T*2^;*TP53*^*Q/Q*^ mice. Tumors were induced by AOM and DSS. Tumor burden was visualized by colonoscopy once per week. Once tumors were established, *Mif* recombination in intestinal epithelial cells were induced by Tamoxifen. Oil-treated mice served as controls. Oil-treated *Mif*^*fl/fl*^;*vilCreER*^*T*2^;*TP53*^*Q/Q*^ mice are abbreviated as *Mif*^*fl/fl*^;*TP53*^*Q/Q*^ and TAM-treated mice as *Mif*^*∆/∆*^;*TP53*^*Q/Q*^. Endpoint analysis at 10 weeks post-AOM. **B** Total tumor number per mouse of oil-treated *Mif*^*fl/fl*^;*TP53*^*Q/Q*^ and TAM-treated *Mif*^*∆/∆*^;*TP53*^*Q/Q*^ mice at 10 weeks post-AOM injection. *Mif*^*fl/fl*^;*TP53*^*Q/Q*^ (oil) mice: *n* = 8; mean = 18.9. *Mif*^*∆/∆*^;*TP53*^*Q/Q*^ (TAM) mice: *n* = 11; mean = 17.8. **C** Macroscopic analysis of tumors per mouse separated in indicated tumor sizes of *Mif*^*fl/fl*^;*TP53*^*Q/Q*^ (oil) and *Mif*^*∆/∆*^;*TP53*^*Q/Q*^ (TAM) mice at endpoint. *Mif*^*fl/fl*^;*TP53*^*Q/Q*^ (oil) mice: *n* = 8; mean = 2.4 (4 mm), 3.3 (3–4 mm), 6 (2–3 mm), 7.3 (2 mm). *Mif*^*∆/∆*^;*TP53*^*Q/Q*^ (TAM) mice: *n* = 11; mean = 0.7 (4 mm), 2.5 (3–4 mm), 6.2 (2–3 mm), 8.4 (2 mm). ns not significant; *p ≤ 0.05. **D** Total tumor sizes of macroscopically analyzed oil-treated *Mif*^*fl/fl*^;*TP53*^*Q/Q*^ and TAM-treated *Mif*^*∆/∆*^;*TP53*^*Q/Q*^ mice at 10 weeks post-AOM. *Mif*^*fl/fl*^;*TP53*^*Q/Q*^ (oil) group: *n* = 151 tumors out of 8 mice; mean = 5; *Mif*^*∆/∆*^;*TP53*^*Q/Q*^ (TAM) group: *n* = 196 tumors out of 11 mice; mean = 3.6. **B**–**D** Student’s *t* test, two sided.

*Mif*^*fl/fl*^;*vilCreER*^*T*2^;*TP53*^*Q/Q*^ mice (named *Mif*^*fl/fl*^*;TP53*^*Q/Q*^) were subjected to AOM/DSS and tumor growth were visualized via colonoscopy (Fig. 3A). At a defined tumor burden, in this *TP53*-engineered CRC model at week 5 after AOM induction, epithelial *Mif* was genetically removed by TAM (abbreviated by *Mif*^*Δ/Δ*^*;TP53*^*Q/Q*^*)*. Controls were oil-treated *Mif*^*fl/fl*^;*vilCreER*^*T*2^;*TP53*^*Q/Q*^ mice, abbreviated by *Mif*^*fl/fl*^*;TP53*^*Q/Q*^ (Fig. 3A). As expected, tumor progression was generally faster in this *TP53*^*R248Q*^ GOF model and tumors reached the defined tumor size to start TAM treatments earlier (5 weeks in *TP53*^*Q/Q*^ mice versus 6 weeks in *TP53*^*Q/+*^ mice) (Fig. 3A compared to Fig. 1A). Moreover, *Mif*^*fl/fl*^*;TP53*^*Q/Q*^ control mice developed more tumors per mouse at their endpoints (mean = 18.9, Fig. 3B) than *Mif*^*fl/fl*^*;TP53*^*Q/+*^ control mice (mean = 15.6, Fig. 1B). In *TP53*^*Q/Q*^ cohorts, the total tumor numbers per mouse had not changed 5 weeks after TAM-induced MIF ablation (Fig. 3B). In contrast, at 5 weeks post TAM, larger tumors were significantly decreased after genetic *Mif* removal, compared to the *Mif*^*fl/fl*^*;TP53*^*Q/Q*^ control group (Fig. 3C). In line, total tumor areas were reduced in MIF-depleted mice (Fig. 3D). We again examined with additional control groups whether Tamoxifen acquired unspecific effects in homozygous *TP53*^*Q/Q*^ cohorts. Here, unfloxed *Mif* alleles (*Mif*^*+l+*^) were combined with *vilCreER*^*T*2^ and *TP53*^*Q/Q*^ alleles (Supplementary Fig. S3). *Mif*^*+/+*^;*vilCreER*^*T*2^;*TP53*^*Q/Q*^ mice (*Mif*^*+/+*^*;TP53*^*Q/Q*^) were also treated with AOM/DSS and oil or TAM as in Fig. 3A. Although there might be a slight but nonsignificant trend of less total tumors per mouse and tumor sizes after MIF ablation (Supplementary Fig. S3A, B), overall tumor areas were unchanged upon TAM treatment (Supplementary Fig. S3C). Thus, we excluded unspecific TAM effects on tumor growth in this *TP53*^*R248Q*^ GOF CRC mouse model.

**Fig. 4:**
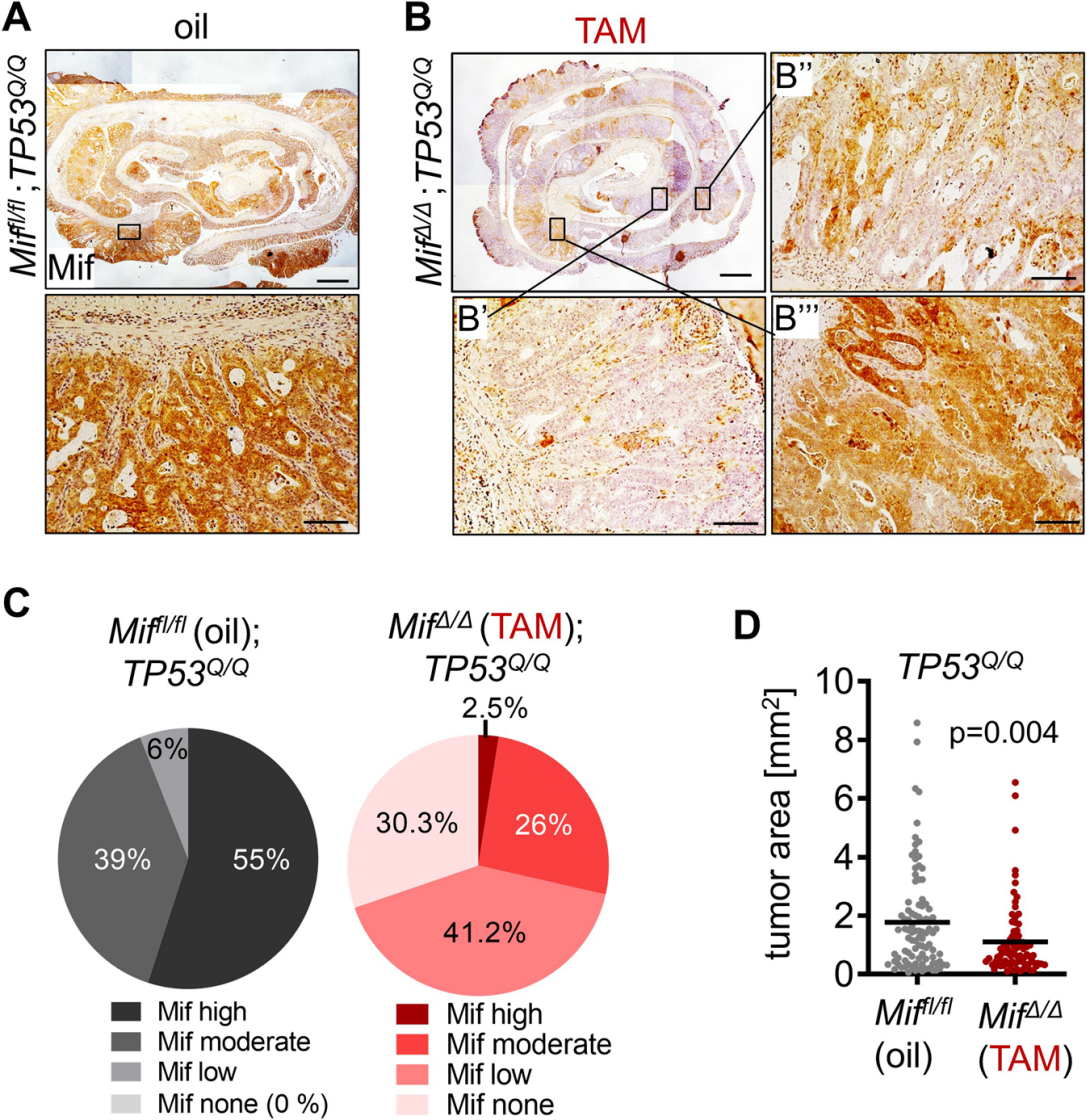
A genetically induced MIF depletion reduces tumor growth in a malignant CRC mouse model. **A** Histological MIF staining in colonic tissues of oil-treated *Mif*^*fl/fl*^;*TP53*^*Q/Q*^ mice at endpoint 10 weeks post-AOM. Representative images. Top, colonic tissue was prepared as swiss roll. Rectangle represents the area of the higher magnification of a tumor (bottom). Scale bar (top), 1000 µm. Scale bar (bottom), 100 µm. **B**–**B”’** Representative histological Mif staining of colonic tissue in TAM-treated *Mif*^*∆/∆*^;*TP53*^*Q/Q*^ mice at endpoint. Top left, overview as swiss roll. Rectangles represent areas of higher magnifications (**B’**–**B”’**). Scale bar, 1000 µm. **B’** No Mif staining in epithelial cells shows efficient recombination. **B”** Low epithelial Mif staining represents sufficient recombination. **B”’** Moderate Mif staining indicates inefficient recombination. **B’–B”’** Scale bars, 100 µm. **C** Quantification of graded Mif staining from (**A**) and (**B**). of all oil-treated *Mif*^*fl/fl*^;*TP53*^*Q/Q*^ (**A**) and all TAM-treated *Mif*^*∆/∆*^;*TP53*^*Q/Q*^ (**B**) swiss roles at endpoint. High and moderate Mif staining represents inefficient recombination. No and low Mif staining represents efficient recombination. Please note, oil-treated *Mif*^*fl/fl*^;*TP53*^*Q/Q*^ mice had no tumors without Mif (Mif none = 0%). *Mif*^*fl/fl*^;*TP53*^*Q/Q*^ (oil) group: *n* = 100 tumors out of 8 mice. *Mif*^*∆/∆*^;*TP53*^*Q/Q*^ (TAM) group: *n* = 119 tumors out of 11 mice. **D** Analysis of tumor areas of all *Mif*^*fl/fl*^;*TP53*^*Q/Q*^ (oil) tumors and all *Mif*^*∆/∆*^;*TP53*^*Q/Q*^ (TAM) tumors graded with efficient *Mif* recombination from (**C**). Tumors areas were measured using ImageJ and calculated as ellipsoid in mm^2^. *Mif*^*fl/fl*^;*TP53*^*Q/Q*^ (oil) group: *n* = 94 tumors out of 8 mice; mean = 1.8. *Mif*^*∆/∆*^;*TP53*^*Q/Q*^ (TAM) group: *n* = 83 tumors out of 11 mice; mean = 1.1. Student’s *t* test, two-sided.

Furthermore, we analyzed *Mif* recombination efficiency as done for heterozygous *TP53*^*Q/+*^ groups. Therefore, we immunostained oil-treated *Mif*^*fl/fl*^*;TP53*^*Q/Q*^ and TAM-treated *Mif*^*Δ/Δ*^*;TP53*^*Q/Q*^ p53 gain-of-function groups for MIF level at 10 weeks endpoint. Again, oil-treated *Mif*^*fl/fl*^*;TP53*^*Q/Q*^ tumors displayed a robust Mif protein stabilization in their tumor epithelial cells (Fig. 4A). Notably, Mif staining in TAM-treated *Mif*^*Δ/Δ*^*;TP53*^*Q/Q*^ tumors displayed a partial *Mif* recombination (Fig. 4B) and were graded in none (Fig. 4B’), low (Fig. 4B”), moderate (Fig. 4B”’) and high Mif level. Again, tumors with insufficient *Mif* recombination (Mif high and Mif moderate) were excluded from microscopic analysis. In detail, in 71.5% of TAM-treated tumors, MIF ablation was grouped in Mif none or low and were processed for further microscopic analysis (Fig. 4C). Tumor areas of the TAM-treated group with a sufficient MIF depletion confirmed the reduction of cancer growth compared to the oil-treated groups (Fig. 4D). Furthermore, possible TAM-induced unspecific effects in unfloxed *Mif*^*+l+*^*;TP53*^*Q/Q*^ cohorts were again excluded (Supplementary Fig. S4A–C). Oil- and TAM-treated *Mif*^*+l+*^*;TP53*^*Q/Q*^ groups, both exposed an intensive Mif staining in tumor epithelial cells (Supplementary Fig. S4A, B). In agreement, TAM treatment in *Mif*^*+l+*^*;TP53*^*Q/Q*^ tumors showed no decreased tumor area compared to oil treatment (Supplementary Fig. S4C) and thus, Tamoxifen have not gained unspecific effects in none of the *TP53*-engineered CRC mouse models.

We demonstrated that *TP53*^R248Q^ GOF mutants promote tumor cell invasion in the CRC AOM/DSS mouse model [58, 59]. Consequently, we analyzed *TP53*^*Q/Q*^ cohorts for invasive tumor cells. Histological analysis of the TAM-treated *Mif*^*Δ/Δ*^*;TP53*^*Q/Q*^ group at the invasive front revealed that both, tumor cells with high MIF level as well as tumor cells without MIF, are able to invade through the muscularis mucosae independent of *Mif* recombination (Supplementary Fig. S4D). In fact, 5 out of 8 of oil-treated *Mif*^*fl/fl*^*;TP53*^*Q/Q*^ mice (63%) harbored one or more invasive tumors, which is comparable to 6 out of 11 TAM-treated *Mif*^*Δ/Δ*^*;TP53*^*Q/Q*^ mice (55%) (Supplementary Fig. S4E). Similarly, calculated in total invasive tumor numbers, a sufficient *Mif* recombination failed to reduce invasive tumor growth (Supplementary Fig. S4F). Moreover, in the human SW480 and DLD-1 CRC cell lines, a MIF depletion via siRNA-mediated *MIF* silencing mainly failed to reduce the migratory capacity of these epithelial tumor cells (Supplementary Fig. S4G, H). The slightly reduced migration in siMIF-depleted DLD-1 cells (Supplementary Fig. S4H) might be originated by reduced viability upon MIF depletion. Accordingly, we tested the viability of siMIF-depleted CRC cells and found that DLD-1 cells, which showed reduced migration, also exhibited decreased viability compared to scramble control (Supplementary Fig. S4I). Thus, MIF promotes other tumorigenic functions than invasion in CRC. In sum, elevated, epithelial MIF is essential for the growth of established tumors and thus, for CRC maintenance in a late-stage CRC mouse model.

### Acute depletion of epithelial MIF reduces angiogenesis and macrophage recruitment in established colorectal tumors

**Fig. 5:**
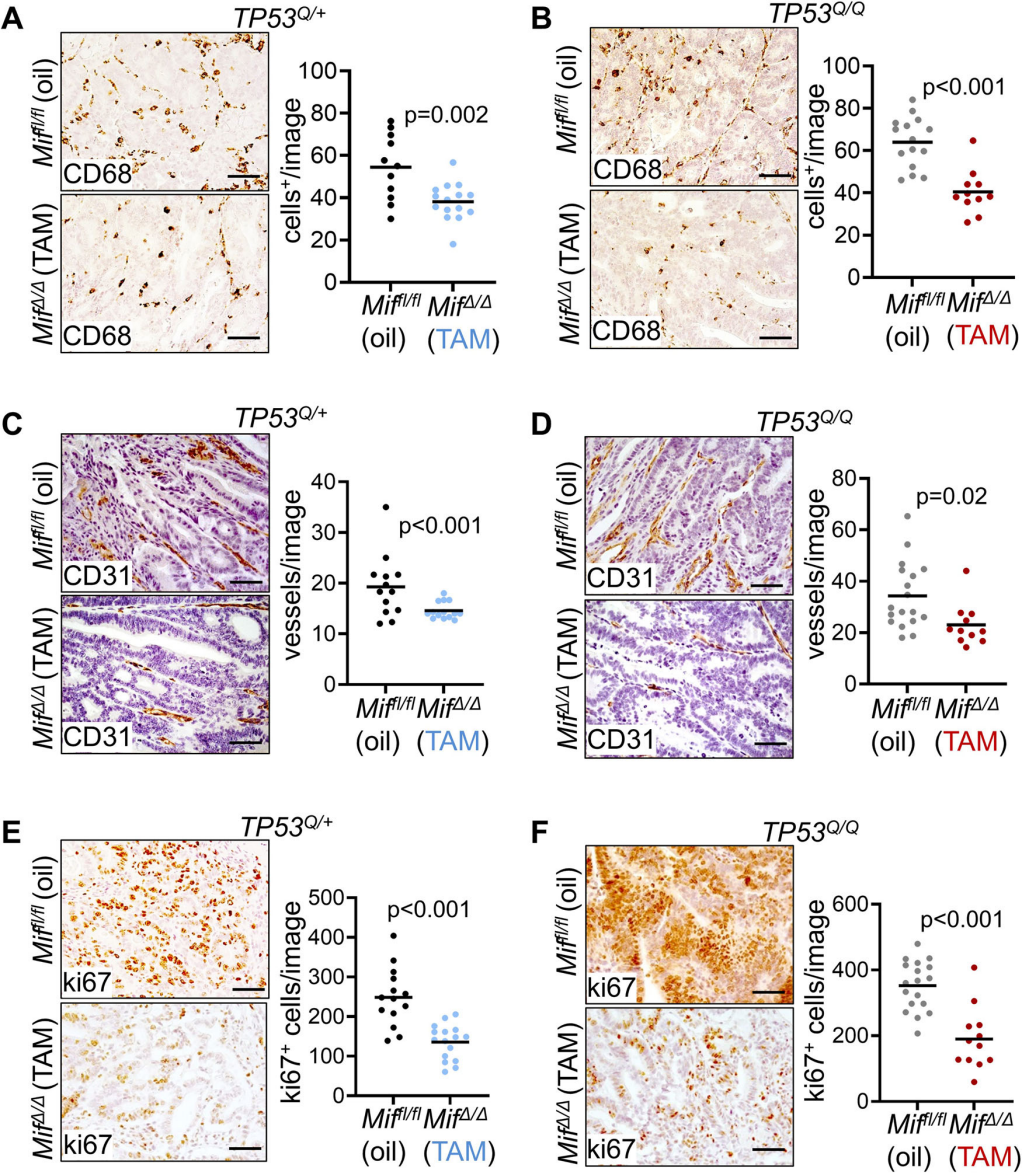
Acute depletion of epithelial MIF reduces macrophage recruitment, angiogenesis and epithelial cell proliferation in established colorectal tumors. **A, C, E** Immunohistological staining and quantifications of CD68 (**A**), CD31 (**C**) and Ki67 (**E**) in oil-treated *Mif*^*fl/fl*^*;TP53*^*Q/+*^ and TAM-treated *Mif*^*∆/∆*^*;TP53*^*Q/+*^ tumors at 12 weeks post-AOM. Right, Quantification of positively stained cells within 2–4 images (×40 magnification) per tumor. **A** *Mif*^*fl/fl*^*;TP53*^*Q/+*^ (oil) group: *n* = 11 out of 5 mice; mean = 54.4. *Mif*^*∆/∆*^*;TP53*^*Q/+*^ (TAM) group: *n* = 15 out of 6 mice; mean = 38.1. **C** *Mif*^*fl/fl*^*;TP53*^*Q/+*^ (oil) group: *n* = 13 out of 5 mice; mean = 19.3. *Mif*^*∆/∆*^*;TP53*^*Q/+*^ (TAM) group: *n* = 14 out of 5 mice; mean = 14.6. **E** *Mif*^*fl/fl*^*;TP 53*^*Q/+*^ (oil) group: *n* = 14 out of 5 mice; mean = 248.6. *Mif*^*∆/∆*^*;TP53*^*Q/+*^ (TAM) group: *n* = 16 out of 6 mice; mean = 135.5. **B**, **D**, **F** Representative staining and quantifications of CD68 (**B**), CD31 (**D**) and Ki67 (**F**) in oil-treated *Mif*^*fl/fl*^*;TP53*^*Q/Q*^ and TAM-treated *Mif*^*∆/∆*^*;TP53*^*Q/Q*^ tumors at 10 weeks post-AOM. Quantifications as in (**A**). **B** *Mif*^*fl/fl*^*;TP53*^*Q/Q*^ (oil) group: *n* = 15 out of 6 mice; mean = 64 *Mif*^*∆/∆*^*;TP53*^*Q/Q*^ (TAM) group: *n* = 11 out of 5 mice; mean = 40.4. **D** *Mif*^*fl/fl*^*;TP53*^*Q/Q*^ (oil) group: *n* = 18 out of 6 mice; mean = 34.3. *Mif*^*∆/∆*^*;TP53*^*Q/Q*^ (TAM) group: *n* = 11 out of 4 mice; mean = 23. **F** *Mif*^*fl/fl*^*;TP53*^*Q/Q*^ (oil) group: *n* = 18 out of 6 mice; mean = 352.2. *Mif*^*∆/∆*^*;TP53*^*Q/Q*^ (TAM) group: *n* = 12 out of 4 mice; mean = 189.7. **A**–**F** Student’s *t* test, two-sided.

It is shown that MIF recruits immune cells and regulates endothelial cell activation, proliferation and endothelial progenitor cell recruitment [11, 14, 41, 46, 70, 71]. Our recent studies in the constitutive MIF knock-out CRC mouse model revealed that MIF supports CRC development via tumor-associated macrophage recruitment and angiogenesis without affecting overall inflammation [18]. Now, we investigated whether these parameters are also affected by *Mif* depletion in established tumors. Therefore, we analyzed the expression of CD68 (a marker of macrophages and monocytes) and CD31 (a vessel marker) at endpoints of the respective *TP53*-engineered CRC mouse models. In both, in *TP53*^*Q/+*^ mice and in *TP53*^*Q/Q*^ GOF mice, infiltration of CD68-positive macrophages was strongly decreased in TAM-treated tumors compared to oil-treated tumors (Fig. 5A, B), supporting the function of MIF as a chemokine to mediate macrophage recruitment [14, 18, 19]. Furthermore, angiogenesis in tumors was reduced after TAM-mediated Mif depletion in both *TP53*-engineered cohorts, as indicated by CD31-positive staining (Fig. 5C, D). However, whether vessel formation is promoted via MIF-regulating pathways in tumor cells themselves (e.g. VEGF expression and secretion) [16, 55, 72] or via VEGF-expressing macrophages [73], or a combination of both, remains elusive. Overall, a Mif depletion in established AOM/DSS-induced tumors strongly affected tumor cell proliferation in both *TP53*-engineered models (Fig. 5E, F), explaining the smaller tumor areas observed in TAM-treated *Mif*^*Δ/Δ*^ cohorts (Figs. 2D and 4D). Importantly, Tamoxifen failed to acquire unspecific effects in both *TP53*-engineered models on the level of tumor-associated macrophage recruitment (Supplementary Fig. S5A, B), vessel formation (Supplementary Fig. S5C, D) and proliferation (Supplementary Fig. S5E, F).

In sum, our therapeutic proof-of-principle CRC mouse models show that tumor-specific stabilized MIF contributes to tumor-associated macrophage recruitment and vessel formation to maintain colorectal tumors, creating a vulnerability which provide a potential targeting strategy in CRC therapies.

## Discussion

For late-stage CRC, novel therapeutic strategies are urgently needed, as 5-year survival rates remain poor - approximately 65% for stage III and only around 10% for stage IV disease. In this study, we identify MIF as a promising drug target in CRC. Utilizing the immune-competent AOM/DSS mouse model, which closely mimics human CRC progression, we selectively depleted epithelial MIF during tumorigenesis at established tumor stages. In both *TP53*-based engineered models, epithelial-specific MIF ablation significantly impaired tumor maintenance. These therapeutic proof-of-principle models highlight the critical role of tumor cell-intrinsic MIF and underscore its potential as a selective and actionable target for anti-cancer therapy in advanced CRC.

Previously, we described a dual role for MIF in CRC using the colitis-associated AOM/DSS mouse model [18]. In that setting, constitutive *Mif* deletion partially protected mice from inflammation-induced tumor initiation, supporting MIF’s established role in host inflammatory responses. However, once tumors were established, MIF-deficient mice lost their overall inflammatory responses and instead, exhibited reduced tumor-associated macrophage recruitment and angiogenesis. This suggests that MIF functions switch from a proinflammatory cytokine to a tumor-specific chemokine and angiogenic-like factor during tumor progression. What remained unclear was whether this reduction in tumor progression resulted from protection during tumor initiation and/or from a functional switch of MIF in established tumors.

To address this, we implemented a time-controlled *Mif* ablation and demonstrated, for the first time in vivo, that CRC tumor maintenance depends on MIF. By inducing *Mif* recombination specifically in intestinal epithelial cells, we showed that tumor growth is significantly reduced - even though stromal MIF remains functionally intact. While stromal cells theoretically contribute minor amounts of MIF to the tumor microenvironment, our data suggest that this source is not sufficient to sustain tumor growth. MIF secreted by stromal cells could bind CD74 on tumor epithelial cells [43, 49, 74], and MIF-deficient stromal cells are known to exhibit reduced functionality [71, 75]. However, in our models, stromal cells such as macrophages are MIF-competent, yet their recruitment is diminished, pointing toward a dominant chemokine function of epithelial-derived MIF. Although it is possible that stromal MIF acts on either stromal or tumor cells, our data clearly demonstrate that epithelial tumor cells are the critical source of MIF required for CRC progression and maintenance.

Importantly, the current model cannot distinguish whether epithelial MIF acts through autocrine and/or paracrine mechanisms. It is well established that MIF can promote an autocrine MIF–CD74 loop in tumor cells to contribute to proliferation and angiogenesis via e.g. VEGF expression [14, 76]. In a paracrine manner, MIF secretion and/or MIF-induced VEGF expression and secretion could directly act on endothelial cells (Fig. 5B) [18, 77, 78]. Furthermore, MIF acts as a chemokine to recruit and polarize macrophages - likely toward an M2 phenotype - further fueling CRC progression [71] (Fig. 5A). These macrophages, in turn, can secrete angiogenic and tumor-supportive factors, creating a feed-forward loop that promotes proliferation and vascularization. Taken together, our findings suggest that MIF-expressing tumor epithelial cells and the recruited macrophages together orchestrate a pro-tumorigenic microenvironment in *TP53*-engineered CRC models. This supports the concept of targeting epithelial MIF as a cancer-selective vulnerability with significant therapeutic potential.

Importantly, the HSP90-mediated stabilization of MIF in epithelial tumor cells is a prerequisite for its tumorigenic activity. We and others have demonstrated that MIF is selectively elevated and stabilized in tumor cells, where it plays a critical role in driving tumor progression [12, 49]. Notably, MIF exhibits pleiotropic functions: it promotes proliferation and inhibits apoptosis across various cancer types, including CRC [14]. In B-cell chronic lymphocytic leukemia (B-CLL), secreted MIF enhances tumor cell survival via activation of the CD74/CD44–IL8 axis and the ERK pathway [79]. Additionally, MIF sustains tumor cell viability through the AKT survival pathway via an autocrine feedback loop [80], and promotes angiogenesis through HIF1α-dependent signaling or the NFκB–IL8–VEGF axis, as reported in several tumor models [77, 81]. MIF can interfere with two major tumor suppressor pathways, p53 and Rb/E2F, that are activated in response to oncogenic signaling [82–84]. Previously, we showed in CRC cell lines that MIF silencing increases p53 activity and p53 target gene expression [20]. Consequently, targeting MIF through pharmacological inhibition of HSP90 represents a promising, tumor-selective strategy.

HSP90 inhibitors have been shown to significantly reduce tumor growth across multiple cancer types [24, 85, 86]. In CRC specifically, HSP90 inhibition not only suppressed tumor growth in vivo, but also selectively impaired the growth of both murine and patient-derived organoids, while leaving organoids from normal epithelial tissue intact [18, 58]. This highlights the therapeutic selectivity of HSP90 inhibitors in targeting MIF-stabilized tumor cells. Moreover, colonic tumor organoids lacking MIF exhibited reduced growth and diminished sensitivity to HSP90 inhibition, supporting the concept that MIF is a critical HSP90 client protein in CRC [18]. Together, these findings confirm that the tumor-specific stabilization of MIF via HSP90 might be often an important driver of CRC progression and represents a selective vulnerability. Thus, elevated MIF level emerges as a viable and promising therapeutic target in CRC.

So far, the availability of potent and druglike MIF inhibitors which are well-characterized in relevant disease models remains limited [4–8]. Most small-molecule MIF inhibitors block its tautomerase activity. However, whether MIF’s enzymatic function is essential for oncogenesis remains an open question [87]. Immune cells appear more responsive to tautomerase inhibition, whereas epithelial tumor cells often exhibit limited sensitivity. This variability may depend on cancer type and the extent to which the immune system promotes tumor progression.

Consequently, tumor-directed MIF inhibitors may provide additional therapeutic opportunities with improved potency and clinical relevance. In line with this, second-generation MIF inhibitors and neutralizing antibodies that directly block MIF protein - rather than just its enzymatic function - are currently under investigation [4–10]. For instance, a novel class of covalent isothiocyanate-based MIF inhibitors demonstrated strong growth-inhibitory effects in colorectal and renal cancer cell lines [88]. Another promising road involves targeting MIF’s homolog, MIF-2 (also known as D-dopachrome tautomerase), the second member of the MIF cytokine family in mammals [10, 89–91]. Both MIF and MIF-2 play critical roles in cancer progression, and their simultaneous inhibition has shown synergistic anti-tumor effects [92, 93]. In addition, strategies have emerged that destabilize MIF protein independently of its enzymatic activity. A MIF-directed proteolysis-targeting chimera (MIF-PROTAC) effectively degraded MIF and suppressed proliferation in lung cancer cells [94]. A particularly innovative approach focuses on targeting oxidized MIF (oxMIF) which is a structural converted MIF isoform in an oxidizing, inflammatory environment [95]. oxMIF is selectively detected in cancerous tissues, including colorectal, pancreatic, and prostate cancers, but not in healthy individuals [96–98]. Thiele and colleagues identified cysteine 81 as a key oxidation site [99], while Skeens et al. used NMR and mass spectrometry to further characterize redox-induced structural changes in MIF [100]. Neutralizing antibodies against oxMIF have shown potential in suppressing pro-tumorigenic functions such as cell proliferation, angiogenesis, and proinflammatory cytokine production [97]. Clinical evaluation of one such antibody, Imalumab, demonstrated good tolerability; however, antitumor activity was modest and may benefit from combination with other therapies [101]. Another promising strategy leverages the tumor specificity of oxMIF antibodies to deliver radionuclides directly to cancer cells, resulting in significant tumor regression in CRC and pancreatic cancer xenograft models [102]. In summary, several novel and selective strategies to inhibit MIF functions - ranging from tautomerase inhibitors to oxMIF-targeting therapies - are under development. While promising, these approaches still require further validation to further establish their clinical relevance in cancer therapy.

The therapeutic potential of targeting MIF is further supported by findings that Mif knockout mice are healthy and fertile, indicating that MIF is dispensable for overall physiological function [82]. This highlights MIF as a tumor-specific vulnerability and suggests that anti-MIF therapies are likely to be well tolerated, with are also shown by minimal toxicity to patients [6, 89, 101, 103]. However, a key challenge in translating MIF inhibition into clinical practice is the current lack of validated drugs that effectively target tumor-relevant MIF functions. Among the most promising approaches might be the indirect depletion of MIF via HSP90 inhibition. In CRC, HSP90 inhibitors are being intensively tested - particularly in combination with chemotherapy and targeted therapies - and are being refined using biomarker-guided strategies based on tumor-specific HSP90 client proteins [104]. We propose that MIF itself serves as such a biomarker in CRC.

Moreover, HSP90 inhibition significantly reduces cancer cell invasion [25, 86]. In contrast, direct MIF depletion alone did not impair invasiveness in our *TP53*-mutant CRC model (Supplementary Fig. S4D–F). This finding aligns with most of the existing literature, which suggests that in CRC, MIF primarily supports tumorigenic functions other than invasion [14, 95]. Where invasive effects were observed in CRC cell lines, they were generally modest [16, 105, 106]. An exception is the murine CRC cell line CT26, in which MIF significantly promotes invasion [107, 108]. Similarly, in other cancers like pancreatic cancer, MIF has been shown to enhance invasion and metastasis [109, 110]. However, in CRC in vivo, the failure of MIF depletion to impact invasion further supports the rationale for using HSP90 inhibitors, which not only induce MIF degradation but also possess inherent anti-invasive activity.

In summary, aberrant MIF level presents a tumor-specific vulnerability that can be exploited therapeutically in CRC. While direct MIF-targeting drugs are not yet clinically available, our proof-of-concept experiments demonstrate that targeting MIF - either directly or via its chaperone machinery - holds significant promise for enhancing anticancer therapy. Moreover, our inducible, tissue-specific CRC models reveal that tumor cell-intrinsic MIF is critical for both tumor progression and maintenance, even in the presence of a fully functional stromal microenvironment. These findings elevate MIF from a mere inflammatory marker to a critical therapeutic target.

## Material & methods

All methods were performed in accordance with the relevant guidelines and regulations.

### Mouse models and genotyping

Animal experiments were approved by state (Niedersächsisches Landesamt für Verbrauchserschutz und Lebensmittelsicherheit, LAVES, Germany) and by the Göttingen University Medical Center Ethik Kommission, ensuring that all experiments conform to the relevant regulatory standards.

All mouse strains have a C57BL/6NCrl background. *Mif*^*flox/flox*^ mice has been described in detail in ref. [82]. In brief, *Mif*^*flox/flox*^ mice, abbreviated as *Mif*^*fl/fl*^, were crossed with *villinCreER*^*T2*^-harboring mice to generate *Mif*^*fl/fl*^*; villinCreER*^*T2*^ mice. In *Mif*^*fl/fl*^;*villinCreER*^*T*2^ engineered mice, floxed MIF alleles can be tissue-specifically recombined through activation of the fusion cre recombinase by Tamoxifen in colonic epithelial tissue. Additionally, mice were crossed to *TP53*^*R248Q*^ alleles [58–60], presented in either a heterozygous (*TP53*^*Q/+*^) or homozygous (*TP53*^*Q/Q*^) *TP53*^*R248Q*^ state. Mice were housed and handled under pathogen-free barrier temperature-controlled (20–22 °C) conditions, with a 12 h day and 12 h dark cycle, with free access to water and standard rodent diet.

DNA isolation and genotyping were performed using DirectPCR lysis Reagent and One-Taq®Quick-Load® Mastermix (New England Biolabs) according to the manufacture’s guidelines. Genotyping Primers are specified in Supplementary Table S1.

### Murine colorectal tumor induction, colonoscopy and treatments

For experiments, randomly selected 8-weeks-old (in a *TP53*^*Q/Q*^ state) and 10-weeks-old (in a *TP53*^*Q/+*^ state) male and female mice weighing at least 20 g were used. Colorectal cancer (CRC) was induced by a single intraperitoneal injection of azoxymethane (AOM, Sigma) at a dose of 10 mg/kg in 0.9% sodium chloride. Following one week rest, 1.7% DSS (*TP53*^*Q/Q*^ background) or 1.9% DSS (*TP53*^*Q/+*^ background) (MP-Biomedicals) was administered via the drinking water for 6 days, to induce acute colitis. The body weights of mice were monitored consistently during and after the AOM/DSS treatment phase.

Tumor development was visualized by mini endoscopy/colonoscopy once per week, starting at week 5. Colonoscopy (Karl Storz GmbH) were performed on anesthetized mice (1.5–2% isoflurane inhalation) meanwhile tumor sizes were assessed based on the method described by Becker & Neurath [111]. In brief, tumor sizes were scored relatively to the colon lumen in S1–S5 grades. S1 = just detectable, S2 = 1/8 of the lumen, S3 = 1/4 of the lumen, S4 = 1/2 of the lumen, S5 > ½ of the lumen. After a defined tumor burden of at least one S2 tumor and ≥three S1 tumors, mice with those established tumors were treated with Tamoxifen (TAM, Sigma-Aldrich) or corn oil as control solution. TAM or oil was given seven times in a row via intraperitoneal injections (1 mg per injection in a 1:10 ethanol/oil mixture) to activate the inducible recombinase (*villinCreER*^*T2*^) to induce MIF allele recombination. Tumor growth was further visualized by colonoscopy once a week. We chose an endpoint analysis, with endpoint at 10 weeks post-AOM in homozygous *TP53*^*Q/Q*^ mice or at 12 weeks post-AOM in p53-heterozygous *TP53*^*Q/+*^ mice.

The endpoint approach minimized the loss of mice caused by colonic obstruction, anal prolapse, or lymphoma development in homozygous *TP53*^*Q/Q*^ mutated mice. For tumor analysis, mice were euthanized, colon and rectum were excised and longitudinally opened. Tumor numbers were counted and tumor sizes were measured using a electronic caliper. Macroscopic total tumor size were calculated using the tumor length and width for ellipsoid formula (A = $\pi \times \text{a}\times \text{b}$) calculation in mm^2^. Afterwards, each colon was “swiss rolled” (inside-out) and fixed in 4% paraformaldehyde/PBS. Using histologic standard processing, samples were formalin-fixed paraffin-embedded (FFPE). Therefore, colonic swiss roles were bisected and placed face down side-by-side into a cassette for histological analysis.

### Histological analysis

Immunohistological stainings were performed with standardized protocols for formalin-fixed paraffin-embedded (FFPE) tissues.

For immunohistological analysis, following primary antibodies were used: MIF (Sigma-Aldrich, HPA003868), Ki67 (Abcam, ab15580), Cluster of differentiation 31 (CD31, Dianova, DIA-310), CD68 (Cell Signaling, 97778) (see Supplementary Table S1). Primary antibodies were detected with ImmPRESS™ Reagent anti-Rabbit IgG (Vector Laboratories). As horseradish peroxidase substrate, 3,3′-Diaminobenzidine tetrahydrochloride (DAB, Roth) were used. Nuclei were counterstained with Hämalaun solution (Merck). Stained sections were imaged by standard microscopy (AxioScope, Zeiss) with ZENblue software V3.0 (Zeiss).

Efficiency of *Mif* recombination was based on Mif staining and grading of each individual tumor bulk. Grading: Mif none = 0% Mif positive cells; Mif low = 1–20% Mif positive cells; Mif moderate = 21–50% Mif positive cells; Mif high ≥ 50% *Mif* positive cells.

Quantification of Ki67, CD31, and CD68 staining were analyzed in a blinded manner. The number of positively stained cells per image were manually counted in 2–4 random fields within one tumor at 40× magnification using CellCounter function in ImageJ software.

Hämalaun & Eosin G (H&E) stained sections were performed to histopathologically analyze swiss roles and to determine invasion of epithelial tumor cells. On H&E-stained sections, tumor areas were microscopically measured using ImageJ software, measuring tumor length and depth followed by calculation based on the ellipsoid formula (A = $\pi \times \text{a}\times \text{b}$). Invasion scoring on H&E stained sections were defined as follow: T0 = tumor cells have not invaded through the muscularis mucosae, T1 = tumor cells invaded into the muscularis mucosae, T2 = tumor cells have invaded through the muscularis mucosae into the submucosa, T3 = cells invade into the muscularis propria. Please note, we have mainly seen T1 and partially T2 stages.

### Cell culture

Human colorectal cancer cell lines SW480 (DSMZ ACC 313) and DLD-1 (DSMZ ACC 278) were grown in RPMI 1604 (Gibco) with 10% FBS, supplemented with Penicillin-Streptomycin (10,000 U/mL, Gibco) and L-Glutamine (Gibco). Cell lines were grown at 37 °C at 5% CO2 in a humidified atmosphere and regularly tested for mycoplasma contamination using the Mycoplasm detection kit (Lonza).

### siRNA transfection, transwell migration assay and cell viability assay

Depletion of human *MIF* was achieved by siRNAs using Lipofectamine™ 2000 (Invitrogen) as transfection reagent. siRNA sequences are listed in Supplementary Table S1. Cells were reverse transfected in 6-well plates (Sarstedt) according to manufacturer guidelines. After 24 h, culture media were re-freshed.

48 h post-transfection, cells were prepared for the transwell migration assays. Therefore, cells were trypsinized and seeded into transwell inserts (Corning) in serum-reduced media (2% FBS). Wells were filled with complete medium. 24 h after seeding, membrane of inserts were fixed in methanol for 10 min and stained with crystal violet (0.1% in 20% EtOH for 20 min). After washing, remaining cells in the insert (on top) were removed using a pre-wet Q-tip, whereas cells that had migrated to the underside were kept and visualized by light microscopy. Migrated cells were counted with ImageJ and calculated relative to scrambled siRNA. For technical replicates cells from one experiment were seeded in two different transwell inserts.

72 h post-transfection, cell viability was assessed using CellTiter-Glo® Luminescent Cell Viability Assay (Promega), according to the manufacture’s’ guidelines. Two in-plate replicates were measured in technical triplicates and viability was normalized to scramble siRNA control.

### Western blots

For protein isolation from human CRC cells, RIPA buffer (1% Triton X-100, 1% sodium deoxycholate, 0.1% SDS, 150 mM NaCl, 10 mM EDTA, 20 mM Tris-HCl pH 7.5, and complete protease inhibitor mix; Roche) was used. Cell lysates were sonicated and centrifuged, and protein concentrations were determined using the BCA protein assay (Pierce). Equal amounts of protein were subjected to SDS-polyacrylamide gel electrophoresis and transferred onto nitrocellulose membranes (Millipore). Membranes were blocked with 5% milk in TBST and incubated with primary antibodies against MIF and β-Actin (Abcam). Details on antibody dilutions are provided in Supplementary Table S1. Densitometric quantification of immunoblot bands was performed using Image Lab^TM^ software (Bio-Rad) and normalized to loading controls.

### Quantification, statistical analysis, and reproducibility

Statistics of each experiment, such as number of animals, number of tumors, precision measures (mean, ±SD), and the statistical tests used for significance are provided in the figures and figure legends. Unpaired (two-sided) Student’s *t* test was used to calculate the *p* values for comparisons of tumor numbers and sizes. Figures were generated wit GraphPad PRISM 9 [version 9.1.0 (221)]. For levels of significance, the following designations were used within this manuscript: *p ≤ 0.05; **p ≤ 0.01; ***p ≤ 0.001; ns not significant.

## Supplementary information

Supplemental Material

## Data Availability

The datasets used and/or analyzed during the current study are available from the corresponding author on reasonable request.

## References

[1] Krebs in Deutschland für 2019/2020. ZENTRUM FÜR KREBSREGISTERDATEN Robert Koch-Institut, Berlin 2023. 2023.

[2] Nopel-Dunnebacke S, Conradi LC, Reinacher-Schick A, Ghadimi M. [Influence of molecular markers on oncological surgery of colorectal cancer]. Chirurg. 2021;92:986–95. 34448902 10.1007/s00104-021-01486-7

[3] Schmitt M, Greten FR. The inflammatory pathogenesis of colorectal cancer. Nat Rev Immunol. 2021;21:653–67. 33911231 10.1038/s41577-021-00534-x

[4] Guo S, Zhao Y, Yuan Y, Liao Y, Jiang X, Wang L, et al. Progress in the development of macrophage migration inhibitory factor small-molecule inhibitors. Eur J Med Chem. 2025;286:117280. 39854942 10.1016/j.ejmech.2025.117280

[5] Kindt N, Journe F, Laurent G, Saussez S. Involvement of macrophage migration inhibitory factor in cancer and novel therapeutic targets. Oncol Lett. 2016;12:2247–53. 27698786 10.3892/ol.2016.4929 PMC5038338

[6] Kok T, Wasiel AA, Cool RH, Melgert BN, Poelarends GJ, Dekker FJ. Small-molecule inhibitors of macrophage migration inhibitory factor (MIF) as an emerging class of therapeutics for immune disorders. Drug Discov Today. 2018;23:1910–8. 29936245 10.1016/j.drudis.2018.06.017 PMC6453109

[7] O’Reilly C, Doroudian M, Mawhinney L, Donnelly SC. Targeting MIF in cancer: therapeutic strategies, current developments, and future opportunities. Med Res Rev. 2016;36:440–60. 26777977 10.1002/med.21385

[8] Sinitski D, Kontos C, Krammer C, Asare Y, Kapurniotu A, Bernhagen J. Macrophage migration inhibitory factor (MIF)-based therapeutic concepts in atherosclerosis and inflammation. Thromb Haemost. 2019;119:553–66. 30716779 10.1055/s-0039-1677803

[9] Khezrian A, Shojaeian A, Khaghani Boroujeni A, Amini R. Therapeutic opportunities in breast cancer by targeting macrophage migration inhibitory factor as a pleiotropic cytokine. Breast Cancer. 2024;18:11782234241276310. 39246383 10.1177/11782234241276310 PMC11380135

[10] Valdez CN, Sanchez-Zuno GA, Bucala R, Tran TT. Macrophage migration inhibitory factor (MIF) and D-dopachrome tautomerase (DDT): pathways to tumorigenesis and therapeutic opportunities. Int J Mol Sci. 2024;25:4849. 10.3390/ijms25094849 PMC11084905 38732068

[11] Nobre CC, de Araujo JM, Fernandes TA, Cobucci RN, Lanza DC, Andrade VS, et al. Macrophage migration inhibitory factor (MIF): biological activities and relation with cancer. Pathol Oncol Res. 2017;23:235–44. 27771887 10.1007/s12253-016-0138-6

[12] Schulz R, Moll UM. Targeting the heat shock protein 90: a rational way to inhibit macrophage migration inhibitory factor function in cancer. Curr Opin Oncol. 2014;26:108–13. 24225413 10.1097/CCO.0000000000000036

[13] Grieb G, Merk M, Bernhagen J, Bucala R. Macrophage migration inhibitory factor (MIF): a promising biomarker. Drug News Perspect. 2010;23:257–64. 20520854 10.1358/dnp.2010.23.4.1453629 PMC3131110

[14] Gordon-Weeks AN, Lim SY, Yuzhalin AE, Jones K, Muschel R. Macrophage migration inhibitory factor: a key cytokine and therapeutic target in colon cancer. Cytokine Growth Factor Rev. 2015;26:451–61. 25882738 10.1016/j.cytogfr.2015.03.002

[15] Mora Barthelmess R, Stijlemans B, Van Ginderachter JA. Hallmarks of cancer affected by the MIF cytokine family. Cancers. 2023;15:395. 10.3390/cancers15020395 PMC9856758 36672343

[16] He XX, Chen K, Yang J, Li XY, Gan HY, Liu CY, et al. Macrophage migration inhibitory factor promotes colorectal cancer. Mol Med. 2009;15:1–10. 19009023 10.2119/molmed.2008.00107 PMC2581606

[17] Wilson JM, Coletta PL, Cuthbert RJ, Scott N, MacLennan K, Hawcroft G, et al. Macrophage migration inhibitory factor promotes intestinal tumorigenesis. Gastroenterology. 2005;129:1485–503. 16285950 10.1053/j.gastro.2005.07.061

[18] Klemke L, De Oliveira T, Witt D, Winkler N, Bohnenberger H, Bucala R, et al. Hsp90-stabilized MIF supports tumor progression via macrophage recruitment and angiogenesis in colorectal cancer. Cell Death Dis. 2021;12:155. 33542244 10.1038/s41419-021-03426-z PMC7862487

[19] Fey RM, Nichols RA, Tran TT, Vandenbark AA, Kulkarni RP. MIF and CD74 as emerging biomarkers for immune checkpoint blockade therapy. Cancers. 2024;16:1773. 10.3390/cancers16091773 PMC11082995 38730725

[20] Schulz R, Marchenko ND, Holembowski L, Fingerle-Rowson G, Pesic M, Zender L, et al. Inhibiting the HSP90 chaperone destabilizes macrophage migration inhibitory factor and thereby inhibits breast tumor progression. J Exp Med. 2012;209:275–89. 22271573 10.1084/jem.20111117 PMC3280870

[21] Schulz R, Streller F, Scheel AH, Ruschoff J, Reinert MC, Dobbelstein M, et al. HER2/ErbB2 activates HSF1 and thereby controls HSP90 clients including MIF in HER2-overexpressing breast cancer. Cell Death Dis. 2014;5:e980. 24384723 10.1038/cddis.2013.508 PMC4040658

[22] Anckar J, Sistonen L. Regulation of HSF1 function in the heat stress response: implications in aging and disease. Annu Rev Biochem. 2011;80:1089–115. 21417720 10.1146/annurev-biochem-060809-095203

[23] Dai C, Whitesell L, Rogers AB, Lindquist S. Heat shock factor 1 is a powerful multifaceted modifier of carcinogenesis. Cell. 2007;130:1005–18. 17889646 10.1016/j.cell.2007.07.020 PMC2586609

[24] Schopf FH, Biebl MM, Buchner J. The HSP90 chaperone machinery. Nat Rev Mol Cell Biol. 2017;18:345–60. 28429788 10.1038/nrm.2017.20

[25] Taipale M, Jarosz DF, Lindquist S. HSP90 at the hub of protein homeostasis: emerging mechanistic insights. Nat Rev Mol Cell Biol. 2010;11:515–28. 20531426 10.1038/nrm2918

[26] Trepel J, Mollapour M, Giaccone G, Neckers L. Targeting the dynamic HSP90 complex in cancer. Nat Rev Cancer. 2010;10:537–49. 20651736 10.1038/nrc2887 PMC6778733

[27] Poggio P, Sorge M, Secli L, Brancaccio M. Extracellular HSP90 machineries build tumor microenvironment and boost cancer progression. Front Cell Dev Biol. 2021;9:735529. 34722515 10.3389/fcell.2021.735529 PMC8551675

[28] Alarcon SV, Mollapour M, Lee MJ, Tsutsumi S, Lee S, Kim YS, et al. Tumor-intrinsic and tumor-extrinsic factors impacting hsp90- targeted therapy. Curr Mol Med. 2012;12:1125–41. 22804236 10.2174/156652412803306729 PMC3521856

[29] Dai C, Sampson SB. HSF1: guardian of proteostasis in cancer. Trends Cell Biol. 2016;26:17–28. 26597576 10.1016/j.tcb.2015.10.011 PMC4722819

[30] Vilaboa N, Bore A, Martin-Saavedra F, Bayford M, Winfield N, Firth-Clark S, et al. New inhibitor targeting human transcription factor HSF1: effects on the heat shock response and tumor cell survival. Nucleic Acids Res. 2017;45:5797–817. 28369544 10.1093/nar/gkx194 PMC5449623

[31] Mendillo ML, Santagata S, Koeva M, Bell GW, Hu R, Tamimi RM, et al. HSF1 drives a transcriptional program distinct from heat shock to support highly malignant human cancers. Cell. 2012;150:549–62. 22863008 10.1016/j.cell.2012.06.031 PMC3438889

[32] Barna J, Csermely P, Vellai T. Roles of heat shock factor 1 beyond the heat shock response. Cell Mol Life Sci. 2018;75:2897–916. 29774376 10.1007/s00018-018-2836-6 PMC11105406

[33] Prince TL, Lang BJ, Guerrero-Gimenez ME, Fernandez-Munoz JM, Ackerman A, Calderwood SK. HSF1: primary factor in molecular chaperone expression and a major contributor to cancer morbidity. Cells. 2020;9:1046. 10.3390/cells9041046 PMC7226471 32331382

[34] Hanahan D, Weinberg RA. Hallmarks of cancer: the next generation. Cell. 2011;144:646–74. 21376230 10.1016/j.cell.2011.02.013

[35] Gomez-Pastor R, Burchfiel ET, Thiele DJ. Regulation of heat shock transcription factors and their roles in physiology and disease. Nat Rev Mol Cell Biol. 2018;19:4–19. 28852220 10.1038/nrm.2017.73 PMC5794010

[36] Talos F, Mena P, Fingerle-Rowson G, Moll U, Petrenko O. MIF loss impairs Myc-induced lymphomagenesis. Cell Death Differ. 2005;12:1319–28. 15947793 10.1038/sj.cdd.4401653

[37] Martin J, Duncan FJ, Keiser T, Shin S, Kusewitt DF, Oberyszyn T, et al. Macrophage migration inhibitory factor (MIF) plays a critical role in pathogenesis of ultraviolet-B (UVB) -induced nonmelanoma skin cancer (NMSC). FASEB J. 2009;23:720–30. 18952710 10.1096/fj.08-119628

[38] Taylor JA 3rd, Kuchel GA, Hegde P, Voznesensky OS, Claffey K, et al. Null mutation for macrophage migration inhibitory factor (MIF) is associated with less aggressive bladder cancer in mice. BMC Cancer. 2007;7:135. 17650334 10.1186/1471-2407-7-135 PMC1939709

[39] Yang S, He P, Wang J, Schetter A, Tang W, Funamizu N, et al. A Novel MIF signaling pathway drives the malignant character of pancreatic cancer by targeting NR3C2. Cancer Res. 2016;76:3838–50. 27197190 10.1158/0008-5472.CAN-15-2841 PMC4930741

[40] Choudhary S, Hegde P, Pruitt JR, Sielecki TM, Choudhary D, Scarpato K, et al. Macrophage migratory inhibitory factor promotes bladder cancer progression via increasing proliferation and angiogenesis. Carcinogenesis. 2013;34:2891–9. 23825153 10.1093/carcin/bgt239 PMC3845890

[41] Asare Y, Schmitt M, Bernhagen J. The vascular biology of macrophage migration inhibitory factor (MIF). Expression and effects in inflammation, atherogenesis and angiogenesis. Thromb Haemost. 2013;109:391–8. 23329140 10.1160/TH12-11-0831

[42] Bach JP, Rinn B, Meyer B, Dodel R, Bacher M. Role of MIF in inflammation and tumorigenesis. Oncology. 2008;75:127–33. 18791328 10.1159/000155223

[43] Mitchell RA, Yaddanapudi K. Stromal-dependent tumor promotion by MIF family members. Cell Sig. 2014;26:2969–78. 10.1016/j.cellsig.2014.09.012 PMC4293307 25277536

[44] Conroy H, Mawhinney L, Donnelly SC. Inflammation and cancer: macrophage migration inhibitory factor (MIF)–the potential missing link. QJM. 2010;103:831–6. 20805118 10.1093/qjmed/hcq148 PMC2955282

[45] Bifulco C, McDaniel K, Leng L, Bucala R. Tumor growth-promoting properties of macrophage migration inhibitory factor. Curr Pharm Des. 2008;14:3790–801. 19128232 10.2174/138161208786898608

[46] Jankauskas SS, Wong DWL, Bucala R, Djudjaj S, Boor P. Evolving complexity of MIF signaling. Cell Signal. 2019;57:76–88. 30682543 10.1016/j.cellsig.2019.01.006

[47] Lee H, Rhee H, Kang HJ, Kim HS, Min BS, Kim NK, et al. Macrophage migration inhibitory factor may be used as an early diagnostic marker in colorectal carcinomas. Am J Clin Pathol. 2008;129:772–9. 18426738 10.1309/GFCLLRH8A68XKMJN

[48] Chen WT, Chang SC, Ke TW, Chiang HC, Tsai FJ, Lo WY. Identification of biomarkers to improve diagnostic sensitivity of sporadic colorectal cancer in patients with low preoperative serum carcinoembryonic antigen by clinical proteomic analysis. Clin Chim Acta. 2011;412:636–41. 21185818 10.1016/j.cca.2010.12.024

[49] Morris KT, Nofchissey RA, Pinchuk IV, Beswick EJ. Chronic macrophage migration inhibitory factor exposure induces mesenchymal epithelial transition and promotes gastric and colon cancers. PLoS One. 2014;9:e98656. 24887129 10.1371/journal.pone.0098656 PMC4041794

[50] Gold DV, Stein R, Burton J, Goldenberg DM. Enhanced expression of CD74 in gastrointestinal cancers and benign tissues. Int J Clin Exp Pathol. 2010;4:1–12. 21228923 PMC3016099

[51] Ramireddy L, Chen WT, Peng CT, Hu RM, Ke TW, Chiang HC, et al. Association between genetic polymorphism of the MIF gene and colorectal cancer in Taiwan. J Clin Lab Anal. 2015;29:268–74. 24840392 10.1002/jcla.21763 PMC6806689

[52] Wang CD, Li TM, Ren ZJ, Ji YL, Zhi LS. Contribution of macrophage migration inhibitory factor -173G/C gene polymorphism to the risk of cancer in Chinese population. Asian Pac J Cancer Prev. 2015;16:4597–601. 26107210 10.7314/apjcp.2015.16.11.4597

[53] Dambacher J, Staudinger T, Seiderer J, Sisic Z, Schnitzler F, Pfennig S, et al. Macrophage migration inhibitory factor (MIF) -173G/C promoter polymorphism influences upper gastrointestinal tract involvement and disease activity in patients with Crohn’s disease. Inflamm Bowel Dis. 2007;13:71–82. 17206642 10.1002/ibd.20008

[54] Shen Y, Guo S, Yang T, Jia L, Chen L, An J, et al. The -173 G/C polymorphism of the MIF gene and inflammatory bowel disease risk: a meta-analysis. Int J Mol Sci. 2013;14:11392–401. 23759989 10.3390/ijms140611392 PMC3709738

[55] Ogawa H, Nishihira J, Sato Y, Kondo M, Takahashi N, Oshima T, et al. An antibody for macrophage migration inhibitory factor suppresses tumour growth and inhibits tumour-associated angiogenesis. Cytokine. 2000;12:309–14. 10805210 10.1006/cyto.1999.0562

[56] Huth S, Huth L, Heise R, Marquardt Y, Lopopolo L, Piecychna M, et al. Macrophage migration inhibitory factor (MIF) and its homolog D-dopachrome tautomerase (D-DT) are significant promotors of UVB- but not chemically induced non-melanoma skin cancer. Sci Rep. 2023;13:11611. 37464010 10.1038/s41598-023-38748-9 PMC10354066

[57] Wirtz TH, Saal A, Bergmann I, Fischer P, Heinrichs D, Brandt EF, et al. Macrophage migration inhibitory factor exerts pro-proliferative and anti-apoptotic effects via CD74 in murine hepatocellular carcinoma. Br J Pharmacol. 2021;178:4452–67. 34250589 10.1111/bph.15622

[58] Schulz-Heddergott R, Stark N, Edmunds SJ, Li J, Conradi LC, Bohnenberger H, et al. Therapeutic ablation of gain-of-function mutant p53 in colorectal cancer inhibits stat3-mediated tumor growth and invasion. Cancer Cell. 2018;34:298–314 e7. 30107178 10.1016/j.ccell.2018.07.004 PMC6582949

[59] Isermann T, Sener OC, Stender A, Klemke L, Winkler N, Neesse A, et al. Suppression of HSF1 activity by wildtype p53 creates a driving force for p53 loss-of-heterozygosity. Nat Commun. 2021;12:4019. 34188043 10.1038/s41467-021-24064-1 PMC8242083

[60] Hanel W, Marchenko N, Xu S, Yu SX, Weng W, Moll U. Two hot spot mutant p53 mouse models display differential gain of function in tumorigenesis. Cell Death Differ. 2013;20:898–909. 23538418 10.1038/cdd.2013.17 PMC3679454

[61] Alexandrova EM, Yallowitz AR, Li D, Xu S, Schulz R, Proia DA, et al. Improving survival by exploiting tumour dependence on stabilized mutant p53 for treatment. Nature. 2015;523:352–6. 26009011 10.1038/nature14430 PMC4506213

[62] Vogelstein B, Kinzler KW. The multistep nature of cancer. Trends Genet. 1993;9:138–41. 8516849 10.1016/0168-9525(93)90209-z

[63] Schwitalla S, Ziegler PK, Horst D, Becker V, Kerle I, Begus-Nahrmann Y, et al. Loss of p53 in enterocytes generates an inflammatory microenvironment enabling invasion and lymph node metastasis of carcinogen-induced colorectal tumors. Cancer Cell. 2013;23:93–106. 23273920 10.1016/j.ccr.2012.11.014

[64] Nakayama M, Sakai E, Echizen K, Yamada Y, Oshima H, Han TS, et al. Intestinal cancer progression by mutant p53 through the acquisition of invasiveness associated with complex glandular formation. Oncogene. 2017;36:5885–96. 28628120 10.1038/onc.2017.194 PMC5658682

[65] Nakayama M, Oshima M. Mutant p53 in colon cancer. J Mol Cell Biol. 2019;11:267–76. 30496442 10.1093/jmcb/mjy075 PMC6487790

[66] Cooks T, Pateras IS, Tarcic O, Solomon H, Schetter AJ, Wilder S, et al. Mutant p53 prolongs NF-kappaB activation and promotes chronic inflammation and inflammation-associated colorectal cancer. Cancer Cell. 2013;23:634–46. 23680148 10.1016/j.ccr.2013.03.022 PMC3657134

[67] Kim MP, Zhang Y, Lozano G. Mutant p53: multiple mechanisms define biologic activity in cancer. Front Oncol. 2015;5:249. 10.3389/fonc.2015.00249 PMC4641161 26618142

[68] Lang GA, Iwakuma T, Suh YA, Liu G, Rao VA, Parant JM, et al. Gain of function of a p53 hot spot mutation in a mouse model of Li-Fraumeni syndrome. Cell. 2004;119:861–72. 15607981 10.1016/j.cell.2004.11.006

[69] Kim MP, Li X, Deng J, Zhang Y, Dai B, Allton KL, et al. Oncogenic KRAS recruits an expansive transcriptional network through mutant p53 to drive pancreatic cancer metastasis. Cancer Discov. 2021;11:2094–111. 33839689 10.1158/2159-8290.CD-20-1228 PMC8338884

[70] Chesney J, Metz C, Bacher M, Peng T, Meinhardt A, Bucala R. An essential role for macrophage migration inhibitory factor (MIF) in angiogenesis and the growth of a murine lymphoma. Mol Med. 1999;5:181–91. 10404515 PMC2230298

[71] Yaddanapudi K, Putty K, Rendon BE, Lamont GJ, Faughn JD, Satoskar A, et al. Control of tumor-associated macrophage alternative activation by macrophage migration inhibitory factor. J Immunol. 2013;190:2984–93. 23390297 10.4049/jimmunol.1201650 PMC3593945

[72] Bozzi F, Mogavero A, Varinelli L, Belfiore A, Manenti G, Caccia C, et al. MIF/CD74 axis is a target for novel therapies in colon carcinomatosis. J Exp Clin Cancer Res. 2017;36:16. 28114961 10.1186/s13046-016-0475-z PMC5260021

[73] Owen JL, Mohamadzadeh M. Macrophages and chemokines as mediators of angiogenesis. Front Physiol. 2013;4:159. 23847541 10.3389/fphys.2013.00159 PMC3701799

[74] Simons D, Grieb G, Hristov M, Pallua N, Weber C, Bernhagen J, et al. Hypoxia-induced endothelial secretion of macrophage migration inhibitory factor and role in endothelial progenitor cell recruitment. J Cell Mol Med. 2011;15:668–78. 20178462 10.1111/j.1582-4934.2010.01041.x PMC3922388

[75] Fan H, Hall P, Santos LL, Gregory JL, Fingerle-Rowson G, Bucala R, et al. Macrophage migration inhibitory factor and CD74 regulate macrophage chemotactic responses via MAPK and Rho GTPase. J Immunol. 2011;186:4915–24. 21411731 10.4049/jimmunol.1003713 PMC3388798

[76] Gaber T, Schellmann S, Erekul KB, Fangradt M, Tykwinska K, Hahne M, et al. Macrophage migration inhibitory factor counterregulates dexamethasone-mediated suppression of hypoxia-inducible factor-1 alpha function and differentially influences human CD4+ T cell proliferation under hypoxia. J Immunol. 2011;186:764–74. 21169549 10.4049/jimmunol.0903421

[77] Winner M, Koong AC, Rendon BE, Zundel W, Mitchell RA. Amplification of tumor hypoxic responses by macrophage migration inhibitory factor-dependent hypoxia-inducible factor stabilization. Cancer Res. 2007;67:186–93. 17210698 10.1158/0008-5472.CAN-06-3292 PMC2941512

[78] Chesney JA, Mitchell RA. 25 years on: a retrospective on migration inhibitory factor in tumor angiogenesis. Mol Med. 2015;21:S19–24. 26605643 10.2119/molmed.2015.00055 PMC4661055

[79] Binsky I, Haran M, Starlets D, Gore Y, Lantner F, Harpaz N, et al. IL-8 secreted in a macrophage migration-inhibitory factor- and CD74-dependent manner regulates B cell chronic lymphocytic leukemia survival. Proc Natl Acad Sci USA. 2007;104:13408–13. 17686984 10.1073/pnas.0701553104 PMC1948950

[80] Lue H, Thiele M, Franz J, Dahl E, Speckgens S, Leng L, et al. Macrophage migration inhibitory factor (MIF) promotes cell survival by activation of the Akt pathway and role for CSN5/JAB1 in the control of autocrine MIF activity. Oncogene. 2007;26:5046–59. 17310986 10.1038/sj.onc.1210318

[81] Kindt N, Lechien JR, Nonclercq D, Laurent G, Saussez S. Involvement of CD74 in head and neck squamous cell carcinomas. J Cancer Res Clin Oncol. 2014;140:937–47. 24663824 10.1007/s00432-014-1648-9 PMC11823928

[82] Fingerle-Rowson G, Petrenko O, Metz CN, Forsthuber TG, Mitchell R, Huss R, et al. The p53-dependent effects of macrophage migration inhibitory factor revealed by gene targeting. Proc Natl Acad Sci USA. 2003;100:9354–9. 12878730 10.1073/pnas.1533295100 PMC170922

[83] Nemajerova A, Mena P, Fingerle-Rowson G, Moll UM, Petrenko O. Impaired DNA damage checkpoint response in MIF-deficient mice. EMBO J. 2007;26:987–97. 17290223 10.1038/sj.emboj.7601564 PMC1852846

[84] Petrenko O, Moll UM. Macrophage migration inhibitory factor MIF interferes with the Rb-E2F pathway. Mol Cell. 2005;17:225–36. 15664192 10.1016/j.molcel.2004.11.052

[85] Miyata Y, Nakamoto H, Neckers L. The therapeutic target Hsp90 and cancer hallmarks. Curr Pharm Des. 2013;19:347–65. 22920906 10.2174/138161213804143725 PMC7553218

[86] Whitesell L, Lindquist SL. HSP90 and the chaperoning of cancer. Nat Rev Cancer. 2005;5:761–72. 16175177 10.1038/nrc1716

[87] Fingerle-Rowson G, Kaleswarapu DR, Schlander C, Kabgani N, Brocks T, Reinart N, et al. A tautomerase-null macrophage migration-inhibitory factor (MIF) gene knock-in mouse model reveals that protein interactions and not enzymatic activity mediate MIF-dependent growth regulation. Mol Cell Biol. 2009;29:1922–32. 19188446 10.1128/MCB.01907-08 PMC2655604

[88] Putha L, Kok LK, Fellner M, Rutledge MT, Gamble AB, Wilbanks SM, et al. Covalent isothiocyanate inhibitors of macrophage migration inhibitory factor as potential colorectal cancer treatments. ChemMedChem. 2024;19:e202400394. 38977403 10.1002/cmdc.202400394

[89] Tilstam PV, Pantouris G, Corman M, Andreoli M, Mahboubi K, Davis G, et al. A selective small-molecule inhibitor of macrophage migration inhibitory factor-2 (MIF-2), a MIF cytokine superfamily member, inhibits MIF-2 biological activity. J Biol Chem. 2019;294:18522–31. 31578280 10.1074/jbc.RA119.009860 PMC6901300

[90] Xiao Z, Osipyan A, Song S, Chen D, Schut RA, van Merkerk R, et al. Thieno[2,3-d]pyrimidine-2,4(1H,3H)-dione derivative inhibits d-dopachrome tautomerase activity and suppresses the proliferation of non-small cell lung cancer cells. J Med Chem. 2022;65:2059–77. 35041425 10.1021/acs.jmedchem.1c01598 PMC8842245

[91] Guo D, Guo J, Yao J, Jiang K, Hu J, Wang B, et al. D-dopachrome tautomerase is over-expressed in pancreatic ductal adenocarcinoma and acts cooperatively with macrophage migration inhibitory factor to promote cancer growth. Int J Cancer. 2016;139:2056–67. 27434219 10.1002/ijc.30278

[92] Merk M, Zierow S, Leng L, Das R, Du X, Schulte W, et al. The D-dopachrome tautomerase (DDT) gene product is a cytokine and functional homolog of macrophage migration inhibitory factor (MIF). Proc Natl Acad Sci USA. 2011;108:E577–85. 21817065 10.1073/pnas.1102941108 PMC3161582

[93] Brock SE, Rendon BE, Xin D, Yaddanapudi K, Mitchell RA. MIF family members cooperatively inhibit p53 expression and activity. PLoS One. 2014;9:e99795. 24932684 10.1371/journal.pone.0099795 PMC4059697

[94] Xiao Z, Song S, Chen D, van Merkerk R, van der Wouden PE, Cool RH, et al. Proteolysis targeting chimera (PROTAC) for macrophage migration inhibitory factor (MIF) has anti-proliferative activity in lung cancer cells. Angew Chem Int Ed Engl. 2021;60:17514–21. 34018657 10.1002/anie.202101864 PMC8362126

[95] Thiele M, Donnelly SC, Mitchell RA. OxMIF: a druggable isoform of macrophage migration inhibitory factor in cancer and inflammatory diseases. J Immunother Cancer. 2022;10:e005475. 10.1136/jitc-2022-005475 PMC9528626 36180072

[96] Thiele M, Kerschbaumer RJ, Tam FW, Volkel D, Douillard P, Schinagl A, et al. Selective targeting of a disease-related conformational isoform of macrophage migration inhibitory factor ameliorates inflammatory conditions. J Immunol. 2015;195:2343–52. 26209628 10.4049/jimmunol.1500572 PMC4543907

[97] Schinagl A, Thiele M, Douillard P, Volkel D, Kenner L, Kazemi Z, et al. Oxidized macrophage migration inhibitory factor is a potential new tissue marker and drug target in cancer. Oncotarget. 2016;7:73486–96. 27636991 10.18632/oncotarget.11970 PMC5341993

[98] Rossmueller G, Mirkina I, Maurer B, Hoeld V, Mayer J, Thiele M, et al. Preclinical evaluation of ON203, a novel bioengineered mAb targeting oxidized macrophage migration inhibitory factor as an anticancer therapeutic. Mol Cancer Ther. 2023;22:555–69. 37067909 10.1158/1535-7163.MCT-22-0676 PMC10157364

[99] Schinagl A, Kerschbaumer RJ, Sabarth N, Douillard P, Scholz P, Voelkel D, et al. Role of the cysteine 81 residue of macrophage migration inhibitory factor as a molecular redox switch. Biochemistry. 2018;57:1523–32. 29412660 10.1021/acs.biochem.7b01156

[100] Skeens E, Gadzuk-Shea M, Shah D, Bhandari V, Schweppe DK, Berlow RB, et al. Redox-dependent structure and dynamics of macrophage migration inhibitory factor reveal sites of latent allostery. Structure. 2022;30:840–50 e6. 35381187 10.1016/j.str.2022.03.007

[101] Mahalingam D, Patel MR, Sachdev JC, Hart LL, Halama N, Ramanathan RK, et al. Phase I study of imalumab (BAX69), a fully human recombinant antioxidized macrophage migration inhibitory factor antibody in advanced solid tumours. Br J Clin Pharmacol. 2020;86:1836–48. 32207164 10.1111/bcp.14289 PMC7444762

[102] Puchol Tarazona AA, Schinagl A, Mirkina I, Rossmueller G, Kerschbaumer RJ, Bachmann F, et al. Pretargeted radioimmunotherapy with the novel anti-oxMIF/HSG bispecific antibody ON105 results in significant tumor regression in murine models of cancer. Mol Cancer Ther. 2024;23:1219–29. 10.1158/1535-7163.MCT-24-0083 PMC11372362 38833646

[103] Jarmula J, Lee J, Lauko A, Rajappa P, Grabowski MM, Dhawan A, et al. Macrophage migration inhibitory factor as a therapeutic target in neuro-oncology: a review. Neurooncol Adv. 2024;6:vdae142. 39233830 10.1093/noajnl/vdae142 PMC11372298

[104] Kryeziu K, Bruun J, Guren TK, Sveen A, Lothe RA. Combination therapies with HSP90 inhibitors against colorectal cancer. Biochim Biophys Acta Rev Cancer. 2019;1871:240–7. 30708039 10.1016/j.bbcan.2019.01.002

[105] Shin HN, Moon HH, Ku JL. Stromal cell-derived factor-1alpha and macrophage migration-inhibitory factor induce metastatic behavior in CXCR4-expressing colon cancer cells. Int J Mol Med. 2012;30:1537–43. 23023114 10.3892/ijmm.2012.1141

[106] Dessein AF, Stechly L, Jonckheere N, Dumont P, Monte D, Leteurtre E, et al. Autocrine induction of invasive and metastatic phenotypes by the MIF-CXCR4 axis in drug-resistant human colon cancer cells. Cancer Res. 2010;70:4644–54. 20460542 10.1158/0008-5472.CAN-09-3828

[107] Wu LH, Xia HH, Ma WQ, Zhong HJ, Xu HX, Wang YM, et al. Macrophage migration inhibitory factor siRNA inhibits hepatic metastases of colorectal cancer cells. Front Biosci. 2017;22:1365–78. 10.2741/4549 28199208

[108] Sun B, Nishihira J, Yoshiki T, Kondo M, Sato Y, Sasaki F, et al. Macrophage migration inhibitory factor promotes tumor invasion and metastasis via the Rho-dependent pathway. Clin Cancer Res. 2005;11:1050–8. 15709171

[109] Funamizu N, Hu C, Lacy C, Schetter A, Zhang G, He P, et al. Macrophage migration inhibitory factor induces epithelial to mesenchymal transition, enhances tumor aggressiveness and predicts clinical outcome in resected pancreatic ductal adenocarcinoma. Int J Cancer. 2013;132:785–94. 22821831 10.1002/ijc.27736 PMC3488363

[110] Sun H, Cheng R, Zhang D, Guo Y, Li F, Li Y, et al. MIF promotes cell invasion by the LRP1-uPAR interaction in pancreatic cancer cells. Front Oncol. 2022;12:1028070. 36703790 10.3389/fonc.2022.1028070 PMC9871987

[111] Becker C, Fantini MC, Wirtz S, Nikolaev A, Kiesslich R, Lehr HA, et al. In vivo imaging of colitis and colon cancer development in mice using high resolution chromoendoscopy. Gut. 2005;54:950–4. 15951540 10.1136/gut.2004.061283 PMC1774595

