# Supplementary material for: Targeting macrophage migration inhibitory factor as a potential therapeutic strategy in colorectal cancer
Source: Oncogenesis. 2025 Aug 20;14(1):30. doi: 10.1038/s41389-025-00572-3 (PMC12368102; doi:10.1038/s41389-025-00572-3)
Supplement: Supplementary file 1 — Supplemental Material [file 41389_2025_572_MOESM1_ESM.pdf]

Supplementary information corresponding to:

Targeting Macrophage Migration Inhibitory Factor as a potential therapeutic strategy in colorectal cancer

Kim Lucia Schneider [1], Luisa Claus [1], Richard Bucala [2] and Ramona Schulz-Heddergott [1,*]

[1] Department of Molecular Oncology, University Medical Center Göttingen, Göttingen, Germany

[2] Departments of Medicine, Pathology, and Epidemiology & Public Health, Yale School of Medicine and Yale Cancer Center, New Haven, CT, USA

* Corresponding author: ramona.schulz-heddergott@med.uni-goettingen.de

Supplementary figures:

Supplemental Figure 1: Tumor analysis of mice with unfloxed Mif alleles in a TP53Q/+ background to determine Tamoxifen unspecific effects in colorectal adenomas.

Supplemental Figure 2: In unfloxed Mif+/+;TP53Q/+ mice, Tamoxifen itself or its induced Cre activation does not reduce colorectal tumor growth.

Supplemental Figure 3: Colorectal carcinoma growth analysis of Tamoxifen-treated constitutive Mif+/+;TP53Q/Q mice.

Supplemental Figure 4: In unfloxed Mif+/+;TP53Q/Q mice, Tamoxifen treatment do not reduce colorectal carcinoma growth. A sufficient Mif recombination in mutant p53 gain-of-function mice do not prevent tumor cell invasion.

Supplemental Figure 5: Macrophage recruitment, angiogenesis and epithelial cell proliferation is not affected in colorectal tumors with unfloxed Mif alleles.

Supplementary Table S1 - Related to Methods: Reagents and Resources

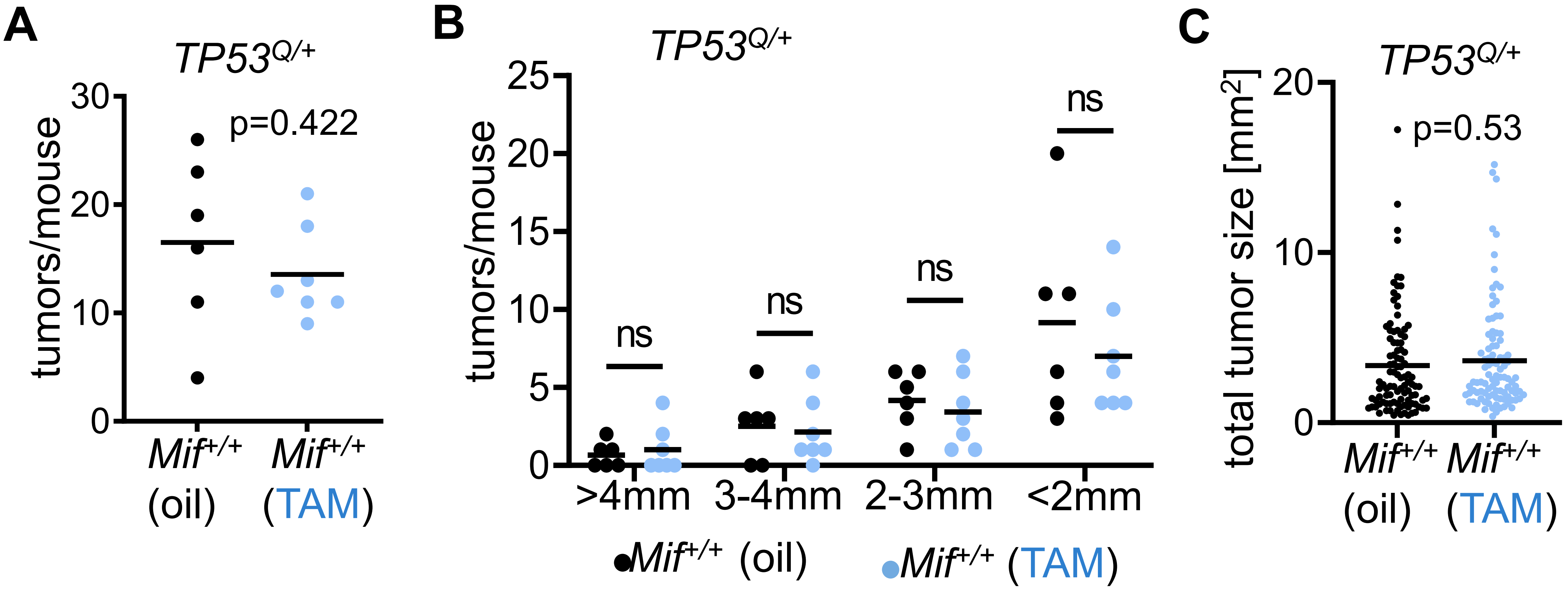

Supp Figure 1

Supplemental Figure 1: Tumor analysis of mice with unfloxed *Mif* alleles in a $TP53^{Q/+}$ background to determine Tamoxifen unspecific effects in colorectal adenomas.

(A) Total tumor numbers per mouse of oil-treated $Mif^{+/+}$;$TP53^{Q/+}$ and TAM-treated $Mif^{+/+}$;$TP53^{Q/+}$ mice at 12 weeks post-AOM injection. $Mif^{+/+}$;$TP53^{Q/+}$ (oil) mice: n = 6; mean = 16.5. $Mif^{+/+}$;$TP53^{Q/+}$ (TAM) mice: n = 7; mean=13.6.

(B) Numbers of indicated tumor sizes per mouse of indicated groups at 12 weeks post-AOM. $Mif^{+/}$;$TP53^{Q/+}$ $^{+}$ (oil) mice: n = 6; mean = 0.7 (4 mm), 2.5 (3-4 mm), 4.2 (2-3 mm), 9.2 (2 mm); $Mif^{+/+}$;$TP53^{Q/+}$ (TAM) mice: n = 7; mean = 1.0 (4 mm), 2.1 (3-4 mm), 3.4 (2-3 mm), 7.0 (2 mm). ns = not significant; $*p \leq 0.05$.

(C) Macroscopic analysis of total tumor sizes of oil-treated $Mif^{+/+}$;$TP53^{Q/+}$ and TAM-treated $Mif^{+/+}$;$TP53^{Q/+}$ mice at 12 weeks post-AOM injection. $Mif^{+/+}$;$TP53^{Q/+}$ (oil) mice: n = 99 tumors out of 6 mice; mean = 3.4. $Mif^{+/+}$;$TP53^{Q/+}$ (TAM) mice: n = 95 tumors out of 7 mice; mean = 3.6.

(A, B, C) Students t-test, two-sided. Black lines, mean.

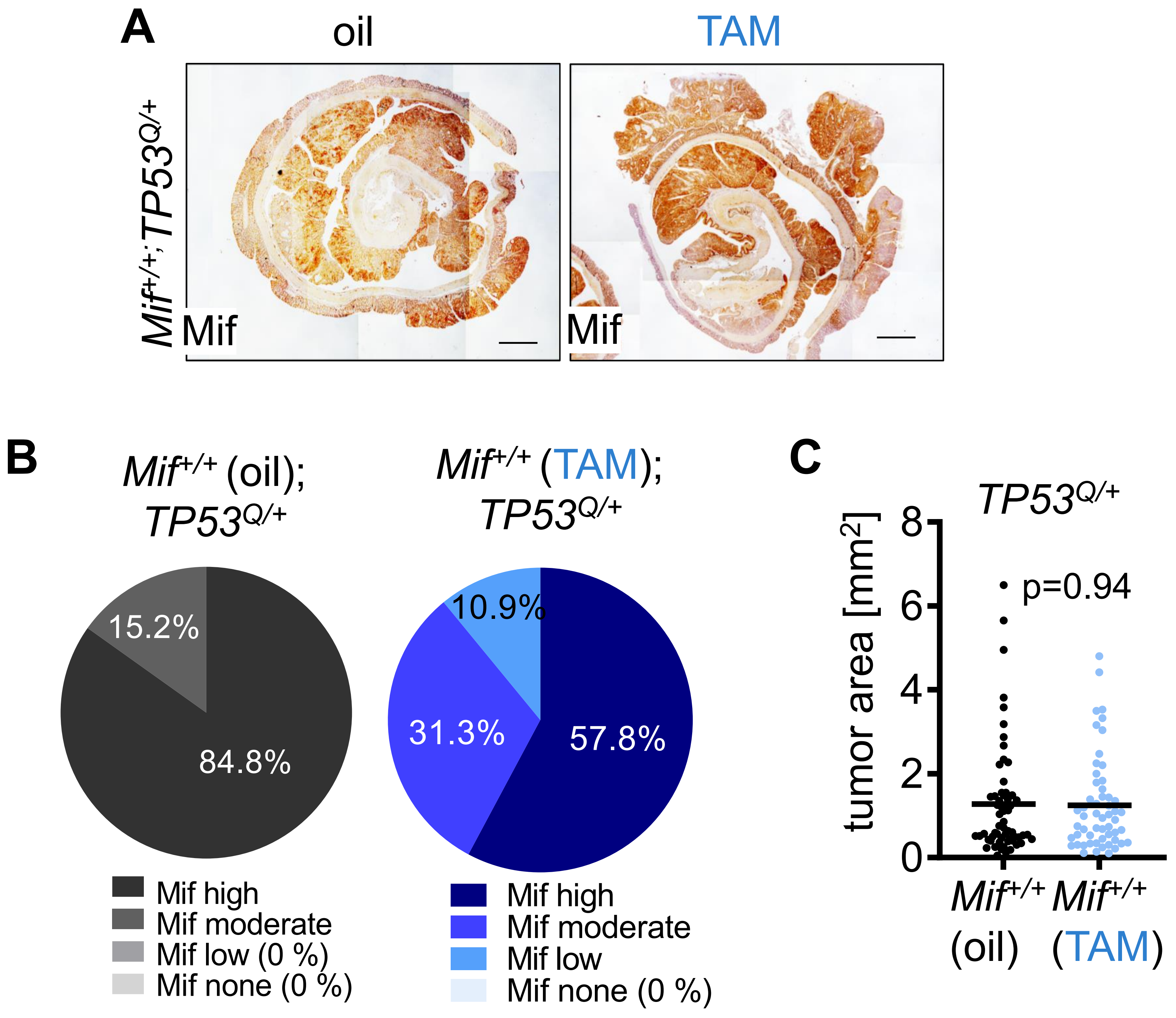

Supp Figure 2

Supplemental Figure 2: In unfloxed *Mif*$^{+/+}$;*TP53*$^{Q/+}$ mice, Tamoxifen itself or its induced Cre activation does not reduce colorectal tumor growth.

(A) Representative immunohistological Mif staining of colonic tissue in oil-treated or TAM-treated *Mif*$^{+/+}$;*TP53*$^{Q/+}$ mice at endpoint 12 weeks post-AOM. Scale bars, 1000 µm.

(B) Quantification of immunhistological Mif staining from (A) of all oil-treated and TAM-treated *Mif*$^{+/+}$;*TP53*$^{Q/+}$ mice at endpoint. Tumors were classified in Mif high, Mif moderate, Mif low and Mif zero, and percentage of tumors per classification were calculated. Please note, all oil-treated *Mif*$^{+/+}$*;TP53*$^{Q/+}$ mice showed moderate or higher Mif protein level. 90% of TAM-treated, unfloxed *Mif*$^{+/+}$*;TP53*$^{Q/+}$ had moderate or higher Mif level. *Mif*$^{+/+}$*;TP53*$^{Q/+}$ (oil) group: n = 66 tumors out of 6 mice. *Mif*$^{+/+}$*;TP53*$^{Q/+}$ group: n = 64 tumors out of 7 mice.

(C) Microscopic analysis of tumor areas of indicated groups at endpoint. Tumors were randomly selected, measured using ImageJ and tumor areas were calculated as ellipsoid in mm^2. *Mif*$^{+/+}$;*TP53*$^{Q/+}$ (oil) mice: n = 61 tumors out of 6 mice; mean = 1.3. *Mif*$^{+/+}$;*TP53*$^{Q/+}$ (TAM) mice: n = 54 tumors out of 7 mice; mean = 1.3. Students t-test, two-sided.

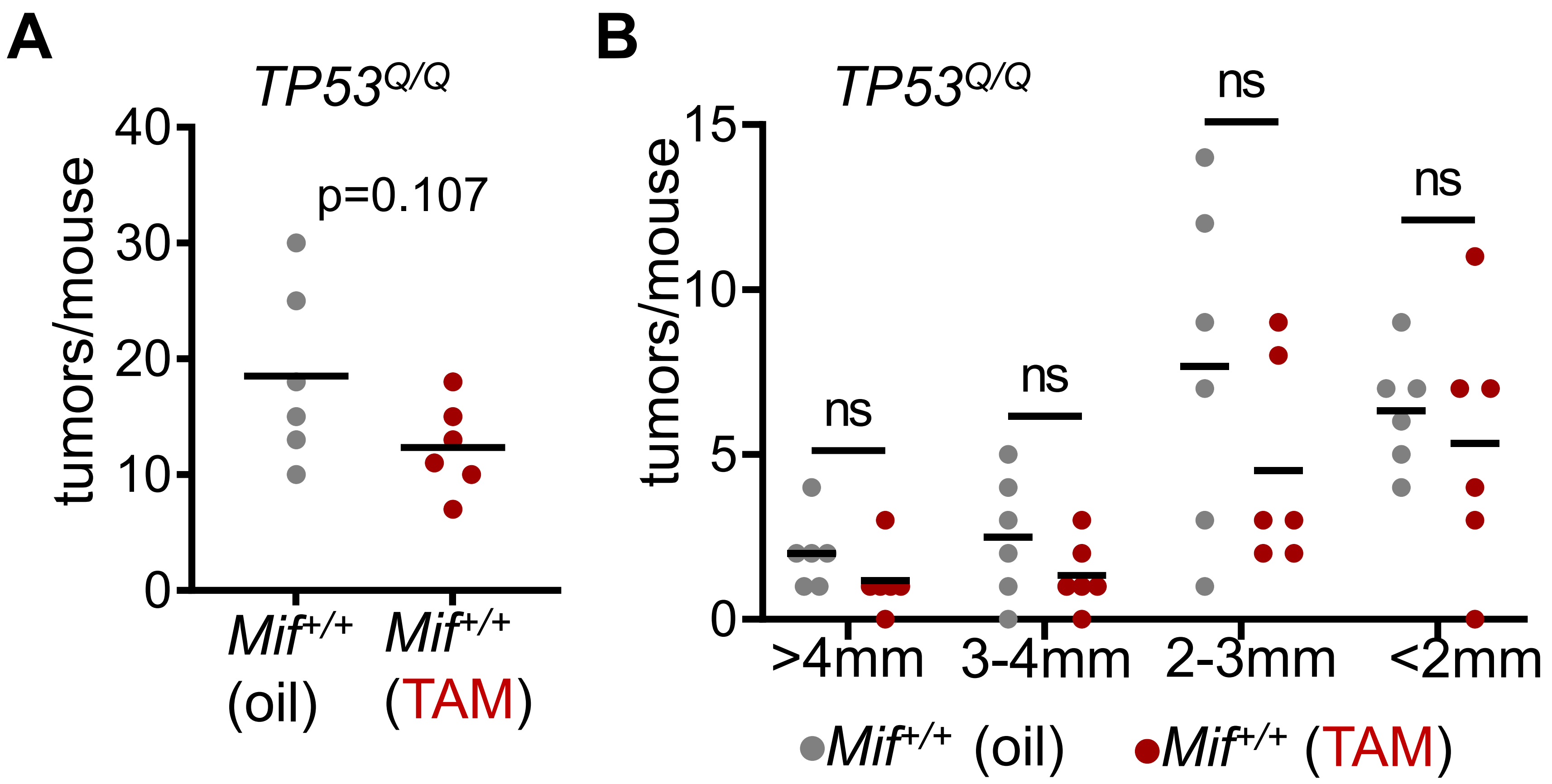

Supp Figure 3

Supplemental Figure 3: Colorectal carcinoma growth analysis of Tamoxifen-treated constitutive *Mif*$^{+/+}$;*TP53*$^{Q/Q}$ mice.

(A) Total tumor numbers per mouse of oil-treated or TAM-treated *Mif*$^{+/+}$;*TP53*$^{Q/Q}$ mice at 10 weeks post-AOM injection. *Mif*$^{+/+}$;*TP53*$^{Q/Q}$ (oil): n = 6; mean = 18.5; *Mif*$^{+/+}$;*TP53*$^{Q/Q}$ (TAM): n = 6; mean = 12.3.

(B) Macroscopic analysis of indicated tumor sizes per mouse of oil-treated or TAM-treated *Mif*$^{+/+}$;*TP53*$^{Q/Q}$ mice at 10 weeks post-AOM. *Mif*$^{+/+}$;*TP53*$^{Q/Q}$ (oil) mice: n = 6; mean = 2.0 (4 mm), 2.5 (3-4 mm), 7.7 (2-3 mm), 6.3 (2 mm). *Mif*$^{+/+}$;*TP53*$^{Q/Q}$ (TAM) mice: n = 6; mean = 1.2 (4 mm), 1.3 (3-4 mm), 4.5 (2-3 mm), 5.3 (2 mm). ns=not significant; *=p ≤ 0.05.

(C) Macroscopic analysis of total tumor sizes of indicated groups at 10 weeks post-AOM. Oil-treated *Mif*$^{+/+}$;*TP53*$^{Q/Q}$ group: n = 111 tumors out of 6 mice; mean = 4.5. TAM-treated *Mif*$^{+/+}$;*TP53*$^{Q/Q}$ group: n = 74 tumors out of 6 mice; mean = 3.8.

(A, B, C) Students t-test, two-sided.

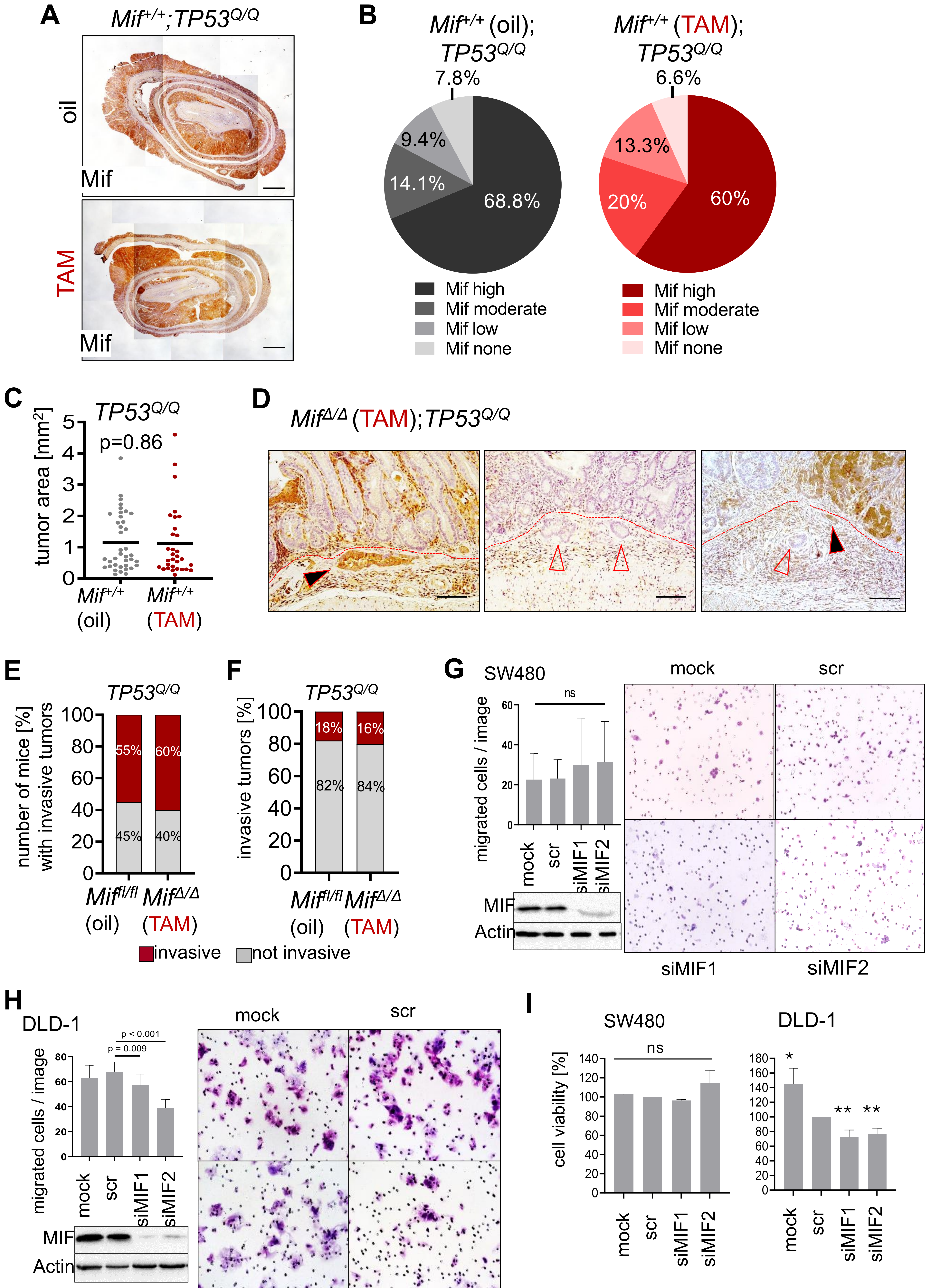

Supp Figure 4

Supplemental Figure 4: In unfloxed *Mif*$^{+/+}$;*TP53*$^{Q/Q}$ mice, Tamoxifen treatment do not reduce colorectal carcinoma growth. A sufficient *Mif* recombination in mutant p53 gain-of-function mice do not prevent tumor cell invasion.

(A) Histological Mif staining in colonic swiss rolls of oil-treated and TAM-treated *Mif*$^{+/+}$;*TP53*$^{Q/Q}$ mice at endpoint week 10 post-AOM. Scale bars, 1000 µm.

(B) Quantification of Mif staining from (A) and classification in indicated groups at endpoint. Tumors were classified in Mif high, Mif moderate, Mif low and Mif zero, and percentage of tumors per classification were calculated. Oil-treated *Mif*$^{+/+}$;*TP53*$^{Q/Q}$ group: n = 64 tumors out of 6 mice. TAM-treated *Mif*$^{+/+}$;*TP53*$^{Q/Q}$ group: n = 45 tumors out of 6 mice.

(C) Tumor area of oil-treated and TAM-treated *Mif*$^{+/+}$;*TP53*$^{Q/Q}$ mice at 10 weeks endpoint. Tumors were randomly selected, measured using ImageJ software and calculated as ellipsoid in mm^2. *Mif*$^{+/+}$;*TP53*$^{Q/Q}$ (oil) n = 38 tumors out of 6 mice; mean = 1.2. *Mif*$^{+/+}$;*TP53*$^{Q/Q}$ (TAM): n = 31 tumors out of 6 mice; mean = 1.1. Students t-test, two-sided.

(D) Histological analysis of *Mif* level in epithelial cells at the invasive front. Three examples of different TAM-treated *Mif*$^{\Delta/\Delta}$;*TP53*$^{Q/Q}$ tumors at endpoint 10 weeks post-TAM. Representative Mif staining. Dashed line, muscularis mucosae. Black filled arrows, invading tumor cells with Mif protein. Red arrows, invading tumors cells without Mif. Scale bars, 100 µm. Please not, tumor epithelial cells are able to invade through the muscularis mucosae into the submucosa independent of Mif protein level.

(E) Number of mice with at least one invasive tumor of calculated as percentage. *Mif*$^{fl/fl}$;*TP53*$^{Q/Q}$ (oil) and *Mif*$^{\Delta/\Delta}$;*TP53*$^{Q/Q}$ (TAM) at 10 weeks post-AOM. If a mouse harbors one or more T1 stage or higher stage tumors, the mouse was positively scored. *Mif*$^{fl/fl}$;*TP53*$^{Q/Q}$ (oil) mice: n = 8. *Mif*$^{\Delta/\Delta}$;*TP53*$^{Q/Q}$ (TAM) mice: n = 11.

(F) Total number of non-invasive and invasive tumors calculated as percentage of oil-treated *Mif*$^{fl/fl}$;*TP53*$^{Q/Q}$ and TAM-treated *Mif*$^{\Delta/\Delta}$;*TP53*$^{Q/Q}$ (TAM) mice at 10 weeks post-AOM. Analysis was done on H&E stained colonic swiss roles. *Mif*$^{fl/fl}$;*TP53*$^{Q/Q}$ (oil) group: n = 100 tumors out of 8 mice. *Mif*$^{\Delta/\Delta}$;*TP53*$^{Q/Q}$ (TAM) group: n = 85 tumors out of 11 mice.

(G, H) Transwell migration assays in SW480 (G) and DLD-1 (H) CRC cell lines with corresponding western blots to analyze MIF silencing efficiency. MIF was depleted with two different siRNAs against *MIF* mRNA. 48 hrs post-transfection, cells were seeded into transwells and were further incubated for 24 hrs. Two technical replicates (n = 2) each. After crystal violet staining of migrated cells, at least four fields per transwell were counted and migrated cells were calculated relative to scrambled controls Mean ± SD. Right, Representative images from the underside of transwells stained with crystal violet. Scale bars, 200 µm. For western blots, MIF-depleted cells of technical in-plate replicates were lysed 72 hrs after transfection and used for immunoblot staining. Actin, loading control.

(I) Cell viability assays of MIF-depleted human SW480 and DLD-1 CRC cells. *MIF* mRNA was silenced as described in (G, H). 72 hrs post-transfection, cells were measured for their viability using the CellTiter-Glo® Luminescent Cell Viability Assay.

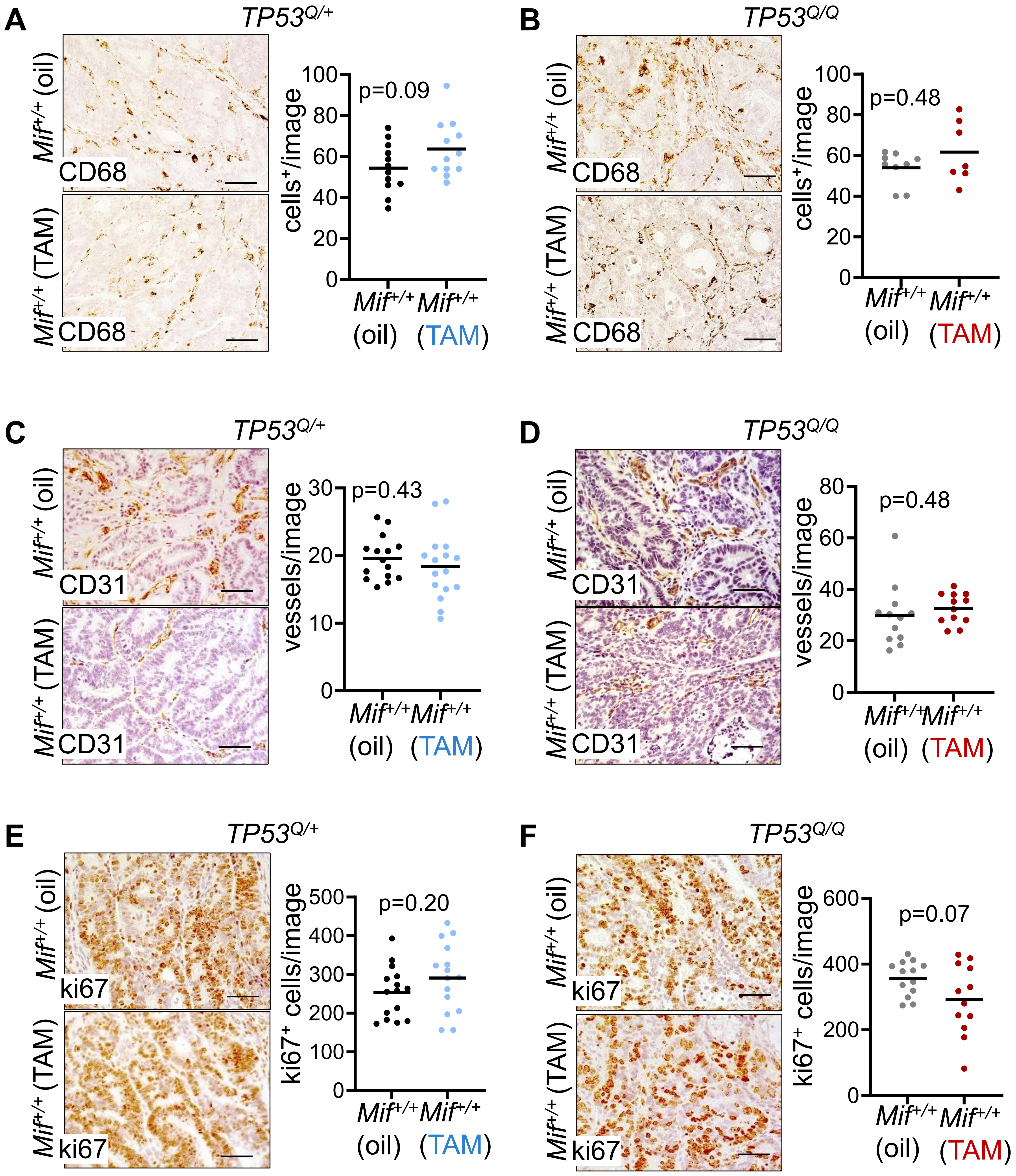
A
TP53Q/+
Mif+/+ (oil)
CD68
Mif+/+ (TAM)
CD68
cells+/image
p=0.09
Mif+/+ (oil)
Mif+/+ (TAM)
B
TP53Q/Q
cells+/image
p=0.48
C
TP53Q/+
CD31
vessels/image
p=0.43
D
TP53Q/Q
p=0.48
E
TP53Q/+
ki67
ki67+ cells/image
p=0.20
F
TP53Q/Q
p=0.07

Supp Fig 5

Supplemental Figure 5: Macrophage recruitment, angiogenesis and epithelial cell proliferation is not affected in colorectal tumors with unfloxed *Mif* alleles.

(A, C, E) Representative stainings and quantifications of CD68 (A), CD31 (C) and Ki67 (E) in oil-treated and TAM-treated $Mif^{+/+};TP53^{Q/+}$ tumors at 12 weeks post-AOM. Right, Quantification of positively stained cells within 2–4 images (×40 magnification) per tumor. **(A)** $Mif^{+/+};TP53^{Q/+}$ (oil) group: n = 12 out of 4 mice; mean = 54.4. $Mif^{+/+};TP53^{Q/+}$ (TAM) group: n = 12 out of 5 mice; mean = 63.7. **(C)** $Mif^{+/+};TP53^{Q/+}$ (oil) group: n = 15 out of 5 mice; mean = 19.6. $Mif^{+/+};TP53^{Q/+}$ (TAM) group: n = 16 out of 6 mice; mean = 18.4. **(E)** $Mif^{+/+};TP53^{Q/+}$ (oil) group: n = 15 out of 5 mice; mean = 254. $Mif^{+/+};TP53^{Q/+}$ (TAM) group: n = 15 out of 5 mice; mean = 291.2.

(B, D, F) Histological stainings and quantifications of CD68 (B), CD31 (D) and Ki67 (F) in oil-treated and TAM-treated $Mif^{+/+};TP53^{Q/Q}$ tumors at 10 weeks post-AOM. Quantifications as in (A). **(B)** $Mif^{+/+};TP53^{Q/Q}$ (oil) group: n = 9 out of 3 mice; mean = 54.0. $Mif^{+/+};TP53^{Q/Q}$ (TAM) group: n = 7 out of 3 mice; mean = 61.7. **(D)** $Mif^{+/+};TP53^{Q/Q}$ (oil) group: n = 12 out of 4 mice; mean = 29.9. $Mif^{+/+};TP53^{Q/Q}$ (TAM) group: n = 12 out of 5 mice; mean = 32.7. **(F)** $Mif^{+/+};TP53^{Q/Q}$ (oil) group: n = 13 out of 4 mice; mean = 357.5. $Mif^{+/+};TP53^{Q/Q}$ (TAM) group: n = 12 out of 4 mice; mean = 292.9.

(A-F) Students t-test, two-sided

Table S1: Related to Methods: Reagents and Resources

Reagent or Resource	Source	Identifier (cat#)
**Antibodies**		
Rat monoclonal anti-CD31	Dianova	DIA-310
Rabbit monoclonal anti-CD68	Cell Signaling	97778
Rabbit polyclonal anti-Ki67	Abcam	ab15580
Rabbit polyclonal anti-MIF	Sigma-Aldrich	HPA003868
ImmPRESS™ Peroxidase polymer reagent Anti-Rat IgG	Vector Laboratories	MP-7444
ImmPRESS™ Peroxidase polymer reagent Anti-Rabbit IgG	Vector Laboratories	MP-7401
Rabbit polyclonal anti-MIF	Sigma-Aldrich HP	HPA003868
Mouse polyclonal anti-beta-Actin	Abcam	ab6276
goat anti-mouse IgG-HRP	Santa Cruz	sc-2005
**Chemicals, Peptides and Recombinant Proteins**		
0.9% sodium chloride	B. Braun	3570310
3,3'-Diaminobenzidine tetrahydrochloride (DAB)	Roth	CN75.2
AOM (Azoxymethane)	Sigma-Aldrich	A5486
Citrate Buffer (10x) Target Retrieval Solution, pH 6.1	Dako Agilent	S169984-2
DirectPCR lysis Reagent	Peqlab	31-101-T
DSS (Dextran sodium sulfate)	MP Biomedicals	160110
Eosin G	Roth	7089.1
Isoflurane CP	CP-Pharma	1214
Mayer's Hemalum solution	Merck	109249
OneTaq® Quick-Load® 2X Master Mix	New England Biolabs	M0486L
Phusion® High-Fidelity DNA Polymerase	ThermoFisher Scientific	F530
Roti ® Histokitt II	Roth	T160.1
Tamoxifen (TAM)	Sigma-Aldrich	T5648
CellTiter-Glo Luminescent Cell Viability	Promega	G7571
**Experimental models: Mouse strains**		
$Mif^{fl/fl}$ (C57BL/6N)	Fingerle-Rowson et al., 2003, Brocks et al., 2017	PMID: 12878730, PMID: 27825106
villin:CreERT2	N/A	RRID:IMSR_JAX:020282
$p53^{R248Q}$	Hanel et al., 2013	PMID: 23538418
**Software and Algorithms**		
GraphPadPRISM®	GraphPad	https://www.graphpad.com
ImageJ software	Open source	https://imagej.net/Welcome
ZEN	Zeiss	https://www.zeiss.de/mikroskopie/produkte/mikroskopsoftware/zen.html
**Genotyping**		
Mif A1	Mouse	5'-AGGTTAGTCACTCTACTGGCC
Mif B1	Mouse	5'-TCTCACTGTTCTGGTGTGAGG
Mif C1	Mouse	5'-GGCTCCTGGTCTCAGTCAGG
Vil Cre	Mouse	5'-CGCGAACATCTTCAGGTTCT
Vil Cre	Mouse	5'-CAAGCCTGGCTCGACGGCC
TP53R248Q = humanized Q	Human/Mouse	5'-AAGGGTGCAGTTATGCCTCA (Human)
Trp53R248Q = WT	Mouse	5'-GGAAGTCCTTTGCCCTGAA 5'-CACTGAAAAAGACCTGGCAACC
**Target of small interfering RNAs**	**Small interfering RNAs**	**Sequence**
Negative control	scr1	undisclosed
MIF	MIF1	5'-CGG ACA GGG UCU ACA UCA-3'
	MIF2	5'-CAA CUC CAC CUU CGC CUA-3'
